# Views of EU citizens on economic growth and implications for climate policy

**DOI:** 10.1038/s41467-026-73323-6

**Published:** 2026-05-19

**Authors:** Ivan Savin, Lewis C. King, Jeroen van den Bergh, Thijs Bouman, Milan Ščasný, E. Keith Smith

**Affiliations:** 1 ESCP Business School, Madrid, Spain; 2 Institute of Environmental Science and Technology, Universitat Autònoma de Barcelona, Bellaterra, Spain; 3 ICREA, Barcelona, Spain; 4 School of Business and Economics & Institute for Environmental Studies, Vrije Universiteit Amsterdam, Amsterdam, The Netherlands; 5 Department of Psychology, University of Groningen, Groningen, The Netherlands; 6 The Environment Centre & Institute of Economic Studies at Faculty of Social Sciences, Charles University, Prague, Czech Republic; 7 Department of Humanities, Social and Political Sciences, ETH Zürich, Zürich, Switzerland

**Keywords:** Decision making, Climate-change policy, Interdisciplinary studies

## Abstract

Although economic growth remains a central objective in policymaking and political discourse, its compatibility with environmental sustainability is increasingly contested. Yet it remains unclear whether this debate is reflected in public opinion. We therefore undertake a cross-national survey of 16,781 people across 13 EU countries to assess citizens’ attitudes in this regard. We find that nearly 60% of citizens express pro-growth views, seeing economic growth as essential for a sustainable society. Of these, more than half hold a moderate and less than half a strong pro-growth view. Additionally, a third of respondents show indifference about growth, while less than 10% hold views that are sceptical about growth. Wealthier countries with less income inequality show lower support for economic growth. Pro-growth attitudes correlate positively with both self-enhancement and self-transcendence values, suggesting that citizens may view growth not only as a means for personal advancement but also as a pathway to collective wellbeing. Growth attitudes have no significant association with climate concern or climate policy support, suggesting a more nuanced picture than a traditional trade-off narrative.

## Abstract (web summary)

Survey results across 13 EU countries show most citizens view economic growth as essential for a sustainable society. No association is found with climate concern or policy support, suggesting a more nuanced picture than a trade-off between goals.

## Introduction

Economic growth has long been viewed as a central mechanism for achieving environmental and social objectives. This assumption underpins the green and inclusive growth strategies promoted by national governments and international institutions such as the World Bank, OECD, and United Nations^1–3^. However, a growing body of literature argues that continued economic growth may face social and environmental limits^4–8^, giving rise to a competing “post-growth” discourse. While recent Eurobarometer data show that citizens identify insufficient economic growth as one of the main challenges facing the EU (19%), social inequalities (21%) and environmental issues are seen as greater concerns (35%)^9^.

Interest in post-growth perspectives has grown rapidly within the research community, as illustrated by a recent surge in both supportive and critical reviews on the topic^10–15^. This trend is also reflected in two recent surveys among climate policy and sustainability researchers, in which more than three-quarters of respondents expressed views that challenge the importance of economic growth^16,17^. Similar discourses have also started to emerge among elected members of the European Parliament^18^. However, it remains unclear if, and to what extent, these debates on pro-growth versus post-growth resonate with the broader public. A better understanding of citizen opinions on these topics is relevant as public attitudes shape electoral outcomes and hence the political feasibility of climate policies^19^.

Post-growth serves as an umbrella term for perspectives that are sceptical of economic growth. At its root are debates over whether growth is desirable for achieving societal objectives and whether it is compatible with biophysical limits. These perspectives are commonly divided into two positions: “agrowth” and “degrowth”. Agrowth adopts an agnostic stance towards growth, arguing that GDP should not be treated as a policy objective as it is not a meaningful indicator of societal progress. Instead, policy should focus directly on achieving environmental and social goals^20^. Degrowth adopts a more critical stance, arguing that growth is neither socially beneficial nor compatible with remaining within planetary boundaries, and therefore calls for an equitable reduction in material consumption^21^.

Previous citizen surveys have typically framed the issue as an implicit trade-off between economic growth and environmental priorities. For instance, recent European-wide and global studies found that around 60% of respondents prioritised the environment when presented with a binary choice between environmental protection and economic growth^22,23^. Other studies have asked respondents to choose between statements representing the main perspectives in the growth-vs-environment literature^24^—“degrowth”, “agrowth”, “green growth” and “growth-at-all-costs”. Studies conducted in 2014 for Spain^25^ and in 2023 for Australia and the UK^26^ indicated that green growth was the dominant position (generally 50–60%). Agrowth (~ 20%) and degrowth (10–15%) had similar distributions across countries, while support for growth-at-all differed more substantially (~ 20% in the UK and Australia, and 4% in Spain).

However, these approaches potentially conflate the perceived trade-offs and prioritisations between economic growth and other objectives with a more fundamental question of whether citizens view economic growth as desirable. Variation in question framing further complicates comparison across contexts, making it difficult to isolate attitudes towards growth itself. To address this, here we move beyond binary choices and instead examine how EU citizens perceive the importance of economic growth for achieving the wider social and environmental objectives of a sustainable society. By focusing on the desirability of growth rather than its trade-offs, we clarify whether citizens view economic growth as a necessary condition to achieve social and environmental goals.

Our analysis addresses three research questions: (1) To what degree do citizens view economic growth as necessary for achieving a sustainable society? (2) How do these perceptions vary across national contexts and individuals’ characteristics and values? (3) What are the potential implications of these perceptions for climate policy support? Rather than asking respondents to choose between economic growth and environmental priorities, and in line with recent literature^27,28^, we assess how necessary citizens perceive economic growth to be for (i) environmental protection, (ii) life satisfaction, (iii) public services, and (iv) economic stability. We address these questions through a survey of 19,328 citizens (16,781 after removing incomplete observations) across 13 EU countries. The EU provides a useful context because it combines diverse economic and social conditions, and it has a well-developed and evolving climate policy. This offers a solid basis to examine how citizens perceive the role of economic growth in relation to climate policy.

Using Latent Class Analysis, we identify four distinct opinion clusters: pro-growth strong, pro-growth moderate, growth-indifferent and growth-sceptical. In addition, we construct a continuous index measuring the intensity of support for, or scepticism towards, economic growth, and examine its correlation with national-level metrics. Through regression analysis, we then assess how both the opinion clusters and the growth support index relate to individuals’ sociodemographic characteristics, individual values, and climate policy preferences. We then discuss how these findings relate to debates about economic growth within academia.

## Results

### Analytical approach

The four statements used in our survey (provided in Table 1) originate from the growth-vs-environment (GEM) instrument^28^. They were selected for their effectiveness in clustering citizens’ views from an original set of 16 survey statements, which were designed to capture the diversity of views in the debate on economic growth. Comparison of Latent Class Analysis outcomes across information criteria indicated that a four-cluster solution was optimal (Supplementary Fig. S1). The distributions of responses to the four GEM survey statements across these clusters are provided in Supplementary Fig. S2.

**Table 1:** Survey statements corresponding to the growth-vs-environment module (GEM)

GEM statement	Survey statement
Environmental protection	Economic growth is necessary to finance environmental protection.
Life satisfaction	Continued economic growth is essential for improving people’s life satisfaction.
Public services	Economic growth is necessary to finance public health and pension systems.
Stability	Without economic growth, the economy will be less stable.

Respondents were asked for their agreement with the statement from strongly disagree to strongly agree on a 7-point Likert scale.

We assigned labels to each cluster based on the median responses to the GEM statements: “pro-growth strong” (25.1%), “pro-growth moderate” (34.7%), “growth-indifferent” (32.1%), and “growth-sceptical” (8.2%). The “pro-growth strong” cluster tended to “strongly agree”, and the “pro-growth moderate” cluster tended to “agree” with all four statements. The distinction between ‘strong’ and ‘moderate’ pro-growth reflects differences in intensity and coherence of support rather than distinct growth paradigms; accordingly, both are treated as sub-types of a broader pro-growth orientation. The “growth-indifferent” cluster tended to “somewhat agree” with the statement on stability and “neither agree nor disagree” with the other three statements. Finally, the “growth-sceptical” cluster tended to either “disagree” or “somewhat disagree”, depending on the statement.

To facilitate statistical comparisons of growth views, we develop a “Growth Support” (GS) index by rescaling the four responses from the Likert scale ranging from 1 to 7 to an equally distanced scale between −1 and +1, as detailed in the Methods section. This index ranks respondents on a scale from −1 (growth-scepticism) to +1 (pro-growth strong). Among the four clusters we identified, growth-scepticism has an average GS value of −0.5, growth-indifferent +0.13, moderate pro-growth +0.58 and strong pro-growth +0.89 (see Supplementary Table S6 for more details). Supplementary Fig. S3 illustrates the overall GS distribution for the full sample.

To examine how growth views relate to sociodemographic characteristics, political views, individual values and policy preferences, we performed two regression analyses: (1) ordinary least squares (OLS) using the GS index, and (2) a multinomial regression comparing opinion clusters relative to the “moderate pro-growth” position as the baseline (see the Methods section for details). The OLS model captures intensity of support or opposition along a single dimension, while multinomial regression identifies qualitatively distinct response profiles that may exhibit non-linear or threshold-like patterns not captured by a single linear scale. Importantly, the clusters are empirically ordered along the GS continuum (with mean GS values increasing monotonically from growth-sceptical to strong pro-growth; see Supplementary Table S6), confirming that they represent structured segments of the same underlying dimension rather than competing conceptualisations.

### Comparison of growth support across European countries

At the national level, we explore how broad economic and social factors relate to citizens’ perceptions of economic growth as a means of achieving a sustainable society. GDP per capita, income inequality, inequality-adjusted Human Development (IHDI) Index and life satisfaction are included to assess whether economic growth is viewed as more of a necessity in contexts where resources are scarce, unevenly distributed, or inefficient at achieving broader well-being. In addition, we consider current greenhouse gas emissions and environmental policy stringency to characterise countries’ current environmental situations and actions.

**Fig. 1:**
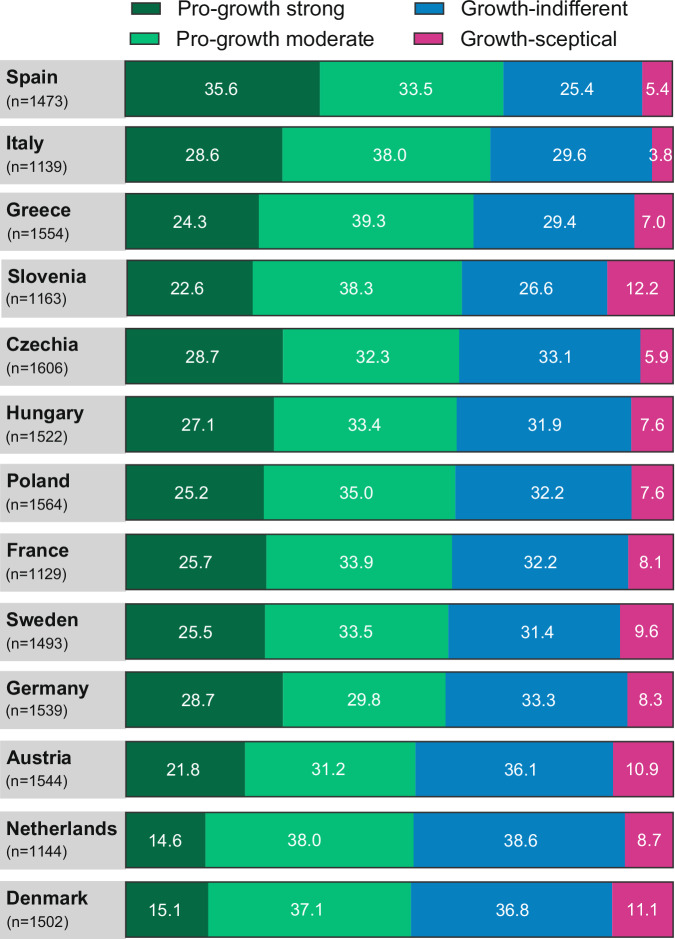
Comparison of growth position clusters across EU countries. Countries are sorted by descending pro-growth views (sum of strong and moderate pro-growth).

Figure 1 shows the distributions of growth opinion clusters across the 13 EU countries included in our study. Spain exhibits the highest proportion of pro-growth views (strong and moderate pro-growth combined) at 69%, while Denmark has the lowest at 52%. In Denmark, as well as the Netherlands and Austria, almost half of the population held either growth-indifferent or growth-sceptical views. Notably, Slovenia displays the highest proportion of growth-sceptical views while ranking the fourth highest when ranked by pro-growth positions.

**Fig. 2:**
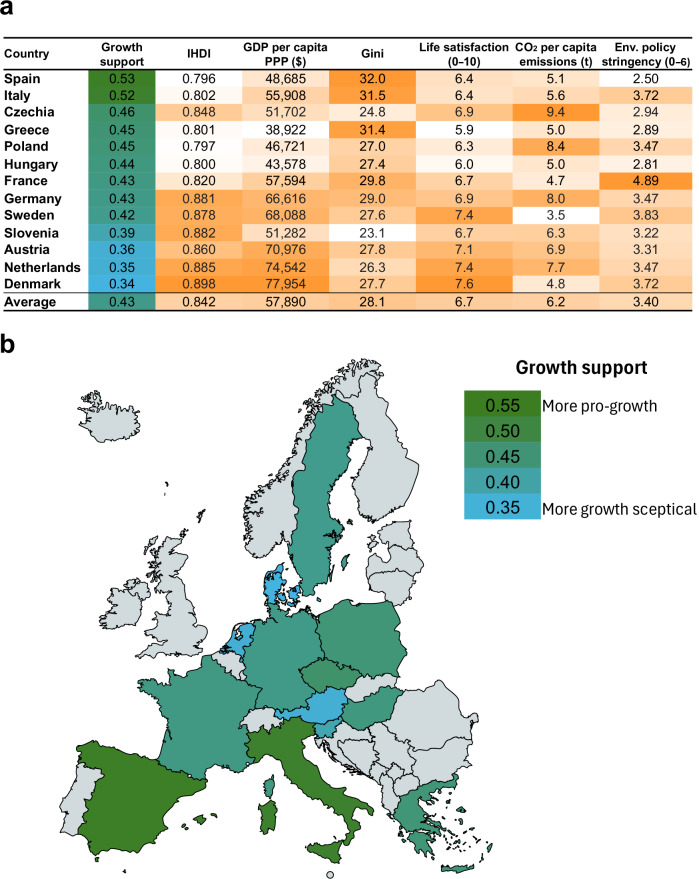
Comparison of growth support (GS) across EU countries. **a** Comparison of growth support to national indicators, including gross domestic product (GDP) per capita at purchasing power parity (PPP), income inequality (Gini coefficient), inequality-adjusted Human Development Index (IHDI), life satisfaction, carbon dioxide (CO₂) emissions per capita, and environmental policy stringency. Sources of data for the national indicators are provided in “Methods”. **b** Map illustrating the average value of the growth support for the countries included in the survey. Darker colours indicate higher values in both (**a**, **b**). Created with mapchart.net. The map uses the Miller cylindrical projection.

Figure 2 presents the average GS values for each of the 13 countries alongside key country-level indicators for the most recent available year: GDP per capita, income inequality (Gini), Inequality-adjusted Human Development Index (IHDI), life satisfaction, CO_2_ emissions per capita, and environmental policy stringency. A correlation matrix between national GS and these country-wide indicators is provided in Supplementary Fig. S4.

A clear pattern emerges for GDP per capita versus income inequality: higher GDP per capita is associated with lower growth support, while greater income inequality (higher Gini) corresponds to higher growth support. Slovenia appears as an outlier in these patterns, given its relatively low GDP per capita and lowest income inequality among the 13 countries. However, when considering IHDI, which combines both income and inequality into one index, Slovenia aligns closely with the broader trend of higher IHDI corresponding to less growth support. As illustrated in Supplementary Fig. S5, among the national indicators, IHDI shows the strongest correlation with GS, with Czechia being the only exception to this trend. The strong association with IHDI may suggest that people living in countries with high income and equality perceive that there are already sufficient resources to achieve other societal goals, such as environmental protection.

Life satisfaction, reflecting citizens’ subjective assessment of their well-being, also correlates with GS, having a magnitude similar to the correlation of GS with GDP per capita. Environmental policy stringency is negatively correlated with growth support, meaning countries with more stringent policies are less likely to hold pro-growth views. In contrast, per capita greenhouse gas emissions show no correlation with growth support, suggesting that current emission levels play little role in shaping citizens’ growth views at the national level.

### Relation to sociodemographics and political views

At the personal level, we first focus on how sociodemographics and political views that are often linked to economic or environmental attitudes relate to perceptions of economic growth as a means to achieve a sustainable society. Sociodemographic characteristics of gender, age, household income, household size and urbanicity are included as these influence exposure to economic insecurity and environmental concerns. Lifestyle indicators of car use and flight frequency serve as proxies for carbon footprints and material consumption.

**Fig. 3:**
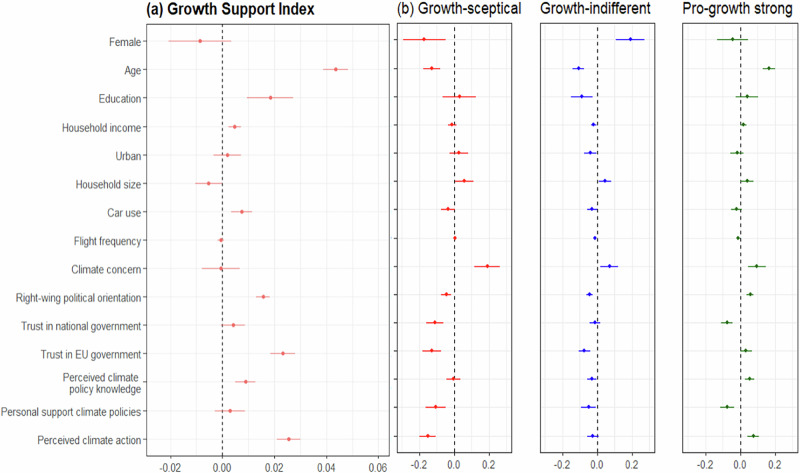
Associations of growth views with sociodemographic characteristics and political views. **a** Coefficient values of OLS regression with the GS index (higher values indicate pro-growth support). **b** Coefficient values of multinomial regression with the moderate pro-growth cluster used as a baseline. In both cases, error bars indicate mean values +/− 2 standard errors. All regressions include country fixed effects (*n* = 16,781).

Political views cover variables related to political orientation, trust in governments, and climate opinions. Political orientation is examined because right-leaning individuals often favour market-based growth solutions^29^, while trust in national and EU governments is considered because institutional trust may affect beliefs about whether growth can be effectively managed to meet social and environmental goals. Finally, we include climate-related variables related to concern, support, perceived knowledge, and perceived action to explore how environmental attitudes align with views on growth as a means to achieve a sustainable society. Figure 3 presents the results, with further detail provided in Supplementary Table S1. Survey statements corresponding to the variables are detailed in Supplementary Table S2.

Among sociodemographic factors, age exhibits a strong relationship with pro-growth views. Older respondents were more likely to have pro-growth views, as reflected in both the GS index and the distribution across opinion clusters. While gender was not statistically significant in the OLS regression, the multinomial regression indicated growth-sceptical respondents were more likely to be male, whereas growth-indifferent respondents were more likely to be female. Higher education levels, household income, and car use were all positively correlated with greater growth support. Flight frequency showed no significant relationship, suggesting that pro-growth attitudes are not necessarily linked to carbon-intensive lifestyle choices.

In terms of political views, right-leaning respondents tend to exhibit stronger support for economic growth, a pattern that is reflected across the opinion clusters, as both growth-indifferent and growth-sceptical clusters were more left-leaning than the moderate pro-growth cluster. Additionally, trust in the EU government (more so than trust in national governments) and perceived climate action by politicians were both positively associated with pro-growth views. Put differently, people less trusting of policymakers, particularly in terms of their action on climate change, are more likely to support alternative views on growth. Perceived climate policy knowledge also showed a slight positive correlation with pro-growth support.

Personal climate concern showed no correlation with the GS index. The multinomial regression reveals that not only growth-sceptical and growth-indifferent clusters but also strong pro-growth were more concerned about climate than moderate pro-growth. There is also no significant correlation between support for climate policies and the GS index, although the moderate pro-growth cluster showed more support than the others. These two findings suggest there is more nuance in citizens’ perceptions of economic growth than seeing it as a simple trade-off between economic and environmental priorities, as often framed in academic discourse and previous citizen surveys^26^.

### Relation to individual values

We now examine how personal values relate to perceptions of economic growth as a means to achieve the broader goals of a sustainable society. While conventional studies imply that those who prioritise environmental protection and social equity would be more sceptical of growth, our measure allows a more nuanced approach by assessing attitudes towards growth without presenting an implicit trade-off. To explore these relationships, we draw on Schwartz’s theory of basic human values.

The survey included a series of statements that correspond to the ten basic individual values developed by Schwartz^30^ (see Supplementary Table S3). These values can be categorised into four higher-order motivational groups: conservation, openness to change, self-enhancement, and self-transcendence. Previous research suggests that self-transcendence and openness to change are positively associated with pro-environmental attitudes, while conservation and self-enhancement are positively associated with anthropocentric concerns such as economic stability^31^. However, given our focus on economic growth’s instrumental role rather than presenting a trade-off, these typical associations may not hold.

**Fig. 4:**
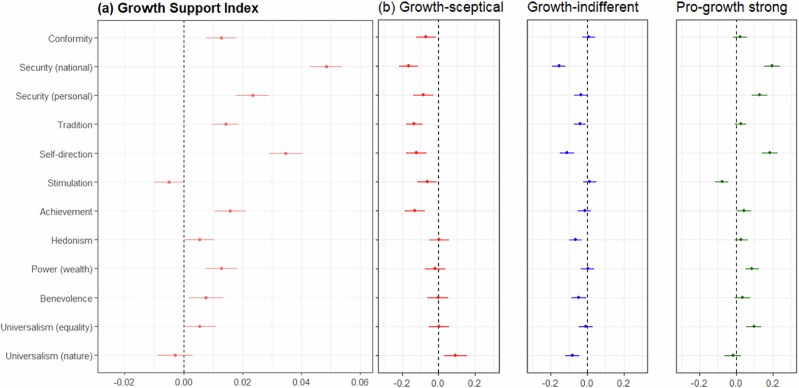
Associations of growth views with basic human values. **a** Coefficient values of the OLS regression with growth support. **b** Coefficient values of multinomial regression with the moderate pro-growth cluster used as a baseline. In both cases, error bars indicate mean values +/− 2 standard errors. All regressions include country fixed effects (*n* = 16,781).

Figure 4a shows the results of the OLS regression, and Fig. 4b displays the multinomial regression outcomes. Values related to “conservation” were positively associated with pro-growth views in both GS scores and opinion clusters. This also aligns with the earlier observation about age, i.e. that pro-growth views are a traditional way of looking at things, whereas more growth-sceptical views are more of an emerging topic that is popular among younger generations and those with greater openness to new ideas. Among these, national security—i.e. living in a secure surrounding, avoiding activities that endanger safety—exhibited the strongest positive correlation. This may reflect that those individuals perceive that changes away from the status quo could endanger their way of living. The two values under “openness to change” produced contrasting results. “Self-direction”, which was framed around independence and autonomous decision-making, showed the second-strongest positive correlation with pro-growth views, following national security. In contrast, “stimulation”, associated with adventure and risk-taking, was negatively associated with pro-growth views. This divergence between “self-direction” and “stimulation” suggests that support for economic growth is not merely a function of openness to change per se but of underlying values. Pro-growth respondents are more likely to hold self-direction values, which may reflect that they see economic growth as a means to achieve independence and control. The association between growth-sceptical respondents and the value of stimulation may reflect their openness to new perspectives and to go against the status quo.

All three values under “self-enhancement”—“achievement”, “power”, and “hedonism”—were positively associated with pro-growth views, aligning with our expectations. Individuals prioritising these values may perceive economic growth either as a reflection of societal success or as a means of personal advancement. Notably, the strong pro-growth cluster showed a positive association with “power”, which was framed around wealth accumulation. This finding is logical as people who endorse these values strongly care about acquiring wealth and possessions, and economic growth serves similar goals at the national level.

In the “self-transcendence” category, both “benevolence” and “equality” were positively associated with pro-growth views, suggesting economic growth is not solely perceived as a pathway for personal gain but also as a means of achieving wider societal well-being. This may indicate that the public does not see economic growth as having limits in terms of achieving social progress, as argued in some of the post-growth literature^6,7,32^. Respect for nature was positively associated with the growth-sceptical cluster and negatively associated with the growth-indifferent cluster, suggesting that a distinguishing factor in those with more sceptical views may be that they place stronger emphasis on the intrinsic value of nature than others.

### Climate policy preferences

We now explore how perceptions of economic growth relate to climate policy preferences. In the survey, we included a series of questions to examine public support for policy instruments that are either existing or proposed at the EU or national level. These policies represent two fundamental approaches to climate action: market-based instruments that rely on economic mechanisms to incentivise emission reductions, and direct regulations that mandate specific behavioural or technological changes. This distinction is potentially relevant to perspectives on economic growth as market-based approaches may be associated with growth-oriented economic paradigms, while regulatory approaches may be perceived as more interventionist and potentially growth-limiting.

The market-based instruments included in the analysis are the existing EU Emissions Trading Scheme (ETS), the proposed ETS for transport, buildings, and agriculture (ETS II); the Carbon Border Adjustment Mechanism (CBAM), an EU rail fund to subsidise rail fares, and taxes on fossil fuel profits, flights, and beef. Direct regulation instruments cover the mandatory (but subsidised for low-income households) insulation of residential buildings, bans on the sales of new internal combustion engine (ICE) vehicles, intensive cattle farming, private jets, and advertising of carbon-intensive goods. Descriptions of how the policy instruments were framed in the survey are provided in Supplementary Table S4.

**Fig. 5:**
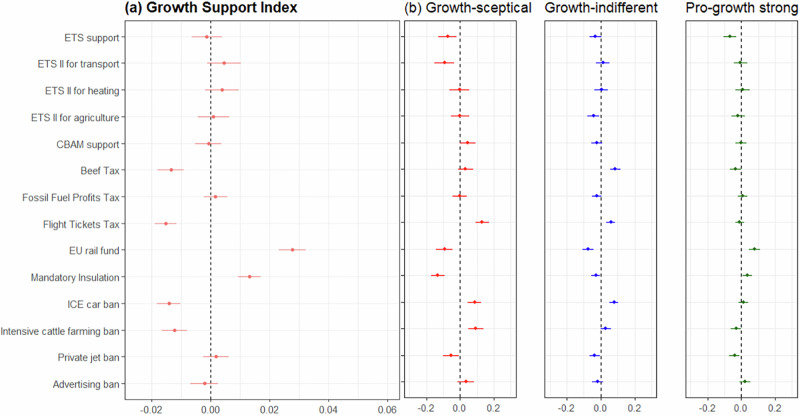
Associations of growth views with support for climate policies. **a** Coefficient values of the OLS regression with growth support. **b** Coefficient values of multinomial regression with the moderate pro-growth cluster used as a baseline. In both cases, error bars indicate mean values +/− 2 standard errors. All regressions include country fixed effects (*n* = 16,781). Policy abbreviations on the *y* axis include emissions trading system (ETS), extension of the emissions trading system to transport, buildings and agriculture (ETS II), carbon border adjustment mechanism (CBAM), and internal combustion engine (ICE).

Figure 5a shows the results of the OLS regression of the Growth Support Index, and Fig. 5b displays the multinomial outcomes for the clusters. Our findings indicate that opinions on ETS and CBAM instruments have little relationship with the overall GS index. However, both growth-sceptical and strong pro-growth are correlated less with general support for ETS, while growth-sceptical respondents show less support for additional ETS for transport.

A clearer divide between the different views emerges with instruments related to taxation. Taxes on beef and flights show negative correlations with the GS index, indicating lower support among pro-growth respondents. In contrast, no significant relationship was found for a tax on fossil fuels. This may indicate that while fossil fuel taxes show balanced support across growth views, those leaning towards growth-sceptical perspectives may be more receptive to policies targeting high-carbon lifestyle choices. In contrast, a subsidised rail fund was more favoured by those with stronger pro-growth views.

Attitudes regarding direct regulation also reveal a clear divide. Growth-indifferent and growth-sceptical respondents show significantly higher support for prohibiting intensive cattle farming and ICE vehicles. However, the results of banning private planes are somewhat contradictory, and there is no clear-cut preference for the advertising of carbon-intensive goods among the four groups. Surprisingly, both growth-indifferent and growth-sceptical clusters show less support for this policy relative to the moderate pro-growth cluster. In contrast, mandatory insulation, which combines direct regulation with targeted subsidies, was positively associated with the GS index, with the growth-sceptical cluster having particularly unfavourable opinions of this instrument. Overall, people who support policies that aim at reducing consumption tend to be more sceptical about growth, whereas those supporting policies that stimulate the economy are typically pro-growth.

## Discussion

Our survey of EU citizens provides evidence on how people view the role of economic growth in achieving the environmental and social goals of a sustainable society across Europe. While a majority of respondents hold pro-growth attitudes, our analysis reveals a complex picture that challenges how aligned the public is with arguments that characterise post-growth literature^14^.

Climate change concern and overall support for climate policies showed no significant relationship with growth views, further indicating that citizens are unlikely to perceive economic growth and sustainability as inherently conflicting. Instead, these findings suggest that people evaluate the role of growth more instrumentally, judging its merit based on perceived necessity for achieving broader societal goals. Moreover, respondents with higher incomes and education levels were more pro-growth, which somewhat contrasts established literature that these groups show more pro-environmental views^33^. In contrast, results were more in line with expectations at the national level. Countries with higher GDP per capita and lower income inequality tend to show less support for economic growth and a shift towards growth-indifferent and sceptical perspectives. This pattern aligns with post-growth arguments that economic growth may become less critical in societies where wealth is already widely distributed and basic needs are met.

Associations with individual values offer insights that arguably are less aligned with expectations. On the one hand, the positive correlation between self-enhancement values and pro-growth views aligns with the conventional interpretation that individuals who prioritise personal success and material accumulation are more likely to view economic growth as desirable. On the other hand, the positive association between self-transcendence values and pro-growth views sketches a more nuanced picture. This may result from people supporting growth because they see it as a means to achieve collective well-being in an equitable and sustainable society rather than solely out of self-interest. This may also explain why pro-growth views appear across the political spectrum and remain predominant among the public, even though most people prioritise self-transcendence over self-enhancement values^33,34^ and support sustainability goals^35^.

In this sense, our findings reveal a fundamental divergence between public and academic perspectives on economic growth. This discrepancy is not merely a difference in opinion but reflects distinct framings of the relationship between growth and sustainability: while academics often frame the debate as a trade-off between growth and environmental protection, citizens appear to perceive growth as a means to an end. Growth may be understood not only instrumentally, as a way to secure personal welfare and stability, but also as a collective good aligned with fairness and prosperity.

The findings further provide insight into how growth perceptions relate to preferences for distinct climate policy. While pro-growth respondents did not systematically favour market-based instruments like ETS and CBAM, they showed strong support for subsidised rail fares and house insulation but low support for taxing carbon-intensive goods, suggesting a preference for direct public investment over carbon pricing mechanisms. Meanwhile, those with growth-indifferent or growth-sceptical views more strongly favoured bans on ICE vehicles and intensive cattle farming, reflecting a preference for firm restrictions on high-carbon consumption. Similarities are also found with the policy preferences of climate policy researchers: in both cases, greater growth scepticism is associated with supporting direct regulation, while those with pro-growth views are more likely to support subsidies^36^.

Our survey of EU citizens suggests that the high level of scepticism towards economic growth observed among climate policy researchers^16,37^ has not spread to the general public, a finding consistent with previous single-country surveys^26,27^. As we explain further in the Supplementary Discussion, EU citizens appear substantially more pro-growth than climate policy researchers. Around 60% of citizens hold pro-growth views, compared with just 14% of researchers. Conversely, only 8% of citizens align with the growth-sceptical position, compared with 35% of academic researchers. However, this comparison should be interpreted with caution because the surveys involve different populations and were conducted several years apart, during which geopolitical and economic developments may have shifted public attitudes. Issues related to security, migration and inflation may therefore contribute to the greater support for economic growth seen in the citizen survey^38,39^.

Nevertheless, the pattern highlights a persistent gap between academic debates on post-growth and the views of the wider public. This gap between public and academic perspectives raises questions about democratic legitimacy and effective climate governance. While post-growth ideas such as agrowth and degrowth are gaining traction in academic debates, they appear to resonate less with the wider public, who continue to lean toward pro-growth positions. This misalignment suggests that either post-growth ideas have not been effectively translated beyond academic circles or their political feasibility is highly limited.

## Methods

### Survey design

From late June to the end of August 2024, we conducted a large-scale, online-based survey in thirteen European countries. The work was carried out under the Horizon Europe “CAPABLE” project,^40^ with the Czech Republic being covered within the SEEPIA programme (No. SS04030013, funded by The Technology Agency of the Czech Republic). In total, 19,328 respondents participated in the survey: Austria (1594), Czechia (1726), Denmark (1591), France (1219), Germany (1597), Greece (1596), Hungary (1593), Italy (1210), the Netherlands (1219), Poland (1595), Slovenia (1201), Spain (1590) and Sweden (1597).

For all countries except the Czech Republic, respondents were recruited through Dynata’s commercial online panel. *National Sample* recruited respondents in the Czech Republic. All respondents were at least 18 years old, and quota sampling was adopted for age (interlocked with gender) and education, matched to the distribution of each national population. Full comparisons between samples and populations for each country are available in Tables S7 and S8 of the Supplementary Materials. For detailed information on data collection of the sample of climate policy researchers, the author is advised to find them in King et al.^16^.

The questionnaire, originally written in English, was translated into each country’s main official language(s) by professional translators and checked by native speakers. On average, the survey took around 22 min to finish. Approval for the study and survey was granted by the ETH Zurich Ethics Committee (EK 2024-N-141).

Several steps were taken to ensure the reliability of responses. These included tracking completion time relative to each country’s median (with a cut-off at 45%), inserting an instruction to choose “other” in a single-choice question, and embedding a similar instruction (“somewhat like me”) in a matrix item. If a respondent failed any two of these checks, their data were excluded, and their place in the sample was refilled. This happened in 6.3% of cases. Median survey lengths, final sample counts, and attention-check failure rates by country are shown in Table S9.

Although the use of commercial, non-probability panels is now widespread in cross-national public opinion research, results should be interpreted with care. Evidence from earlier work suggests that such panels can produce consistent and credible estimates^41–43^. However, as with all convenience-based samples, the absence of random sampling limits the generalisability of the findings and should therefore be interpreted with appropriate caution^44^.

The survey included four statements from the concise ‘Growth-vs-Environment’ Module^19^ to categorise respondents based on their growth-versus-environment opinions. Respondents had to indicate their level of agreement using a Likert scale from 1 (strongly disagree) to 7 (strongly agree): (1) ‘Economic growth is necessary to finance environmental protection’ (Environmental protection), (2) ‘Continued economic growth is essential for improving people’s life satisfaction’ (Life satisfaction), (3) ‘Economic growth is necessary to finance public health and pension systems’ (Public services) and (4) ‘Without economic growth the economy will become less stable’ (Stability). Participants could select 'don’t know' for each statement, and respondents who chose this for at least one of the four statements were excluded from the final sample.

These four statements were taken from Drews et al.^27^ who originally used 16 statements on distinct samples of the general public and international academic researchers, demonstrating that segmentation of respondents into distinct opinion clusters based on many dimensions of the growth debate is more accurate than asking people to choose one of the distinct views. The statements were chosen based on their power to predict the opinion clusters resulting from using all 16 statements to strike a trade-off between brevity and accuracy. The authors also showed that the clustering results produced based on these items have a strong positive correlation with responses to the direct question regarding preferred position in the growth-vs-environment debate, thus validating the items both for the general public and for the experts.

Savin et al.^28^ later showed that one can reduce the 16 statements to just 3–4 while maintaining high predictive accuracy while achieving a concise survey module. While the original GEM analysis suggested that different questions were optimal to use depending on whether the survey was aimed at academics or citizens, two statements were consistent across both groups: (1) “Economic growth is necessary to fund environmental protection” and (2) “Continued economic growth is essential to improve people’s life satisfaction”. These two consistent questions are used for the comparison made between the two groups in Fig. S5.

As participants had the option not to answer the GEM questions, we lost 956 participants due to missing observations, which is less than 5% of our sample. In addition, we lost 63 observations from survey respondents choosing the “other non-binary” option for gender and 1534 observations due to respondents not wanting to report their income. Thus, the sample used in regression analysis covers 16,781 observations.

Our survey also contained a set of questions regarding support for various climate policy instruments such as the EU ETS, CBAM, ban on selling petro/diesel cars, as well as a set of questions on beliefs and values of the respondents and their sociodemographic characteristics (age, gender, household size, income, etc.). These questions are detailed in Tables S2–S5 of the Supplementary Materials.

GDP per capita PPP data were obtained from the World Bank^45^. Inequality-adjusted Human Development Index data were obtained from the United Nations Development Programme^46^. Gini coefficients and Life Satisfaction data were obtained from Eurostat^47^. Carbon dioxide emissions per capita data were obtained from the Emissions Database for Global Atmospheric Research (EDGAR) database^48^. The environmental policy stringency (EPS) index was obtained from the OECD^49^. All data years are for 2022 except for the EPS index, for which 2020 was the most recent data point.

### Clustering of respondents and devising a composite indicator of growth support

We used Latent Class Analysis to segment our sample into four clusters (Fig. S1 in Supplementary Information indicates four clusters as optimal), corresponding to strong and moderate pro-growth, growth-indifferent and growth-sceptical perspectives. Distributions of each clusters’ responses to the four GEM questions are shown in Supplementary Fig. S2. The strong pro-growth cluster tended to “strongly agree” with the statements, the moderate pro-growth cluster “agree”, the growth-indifferent cluster “neither agree nor disagree”, and the growth-sceptical cluster “disagree”.

We derived a Growth Support (GS) Index for each respondent to use in the regression analysis. To this end, we rescaled the four responses from the Likert scale ranging from 1 to 7 to an equally distanced scale between −1 and +1 (−1, −2/3, −1/3, 0, 1/3, 2/3, 1). Then we took the arithmetic average of these four rescaled responses, resulting in an index in the range of −1 to +1 (see Fig. S3).

### Regression analysis

We employed ordinary least squares in the case of the GS Index and multinomial regression models for the four clusters to study the association of growth positions with values/beliefs, sociodemographics, and climate policy preferences. The OLS model with the GS Index collapses the four opinion clusters into a single dimension and captures overall tendencies. The multinomial regression instead compares the probability of belonging to one of the three opinion clusters relative to the baseline category (“moderate pro-growth”). This allows us to identify how covariates shift respondents between specific positions, but it does not assume a simple linear continuum.

In running the regression models, we simultaneously included these three groups of control variables on the right-hand side of the equation, while presenting results of those regressions in the main text in blocks. This has the advantage that we minimise the omitted variable bias. We also considered regressing the resulting clusters separately on the three groups of explanatory variables (see Tables S11–S13 in the Supplementary Information). When variables like trust and climate concern are omitted, some policy measures (e.g., ETS for transport and heating) appear significant, demonstrating that variation in policy preferences is partly explained by these underlying characteristics and the value from pooling all variables in one model. We also estimated the models separately for each country in our dataset (see Table S14 for results for Denmark, Germany and Spain). The country-specific estimates are broadly consistent with the pooled results, with some variation in coefficient magnitudes and statistical significance, but no systematic departures from the overall patterns.

Undertaking Variance Inflation Factor (VIF) analysis indicates that there is no multicollinearity, as all individual values are well below the conservative threshold of 5. To further limit the risk of model overfitting, we used the Least Absolute Shrinkage and Selection Operator (LASSO)^50,51^. The results presented in Table S10 confirm that the key covariates highlighted in the discussion remain stable when penalising less informative predictors.

Note that since our survey data is non-experimental and cross-sectional, we can only derive associational or correlational results, which should not be interpreted as causal statements.

### Reporting summary

Further information on research design is available in the Nature Portfolio Reporting Summary linked to this article.

## Supplementary information

Supplementary Information

Reporting Summary

Transparent Peer Review file

## Data Availability

The data collection and research project were preregistered at OSF. Full replication data are available from GitHub at 10.5281/zenodo.19666333.

## Code availability

The full replication analytical code is archived alongside the replication data and available from GitHub at 10.5281/zenodo.19666333. We used R version 4.4.1.
