# Supplementary material for: Views of EU citizens on economic growth and implications for climate policy
Source: Nat Commun. 2026 May 19;17:6580. doi: 10.1038/s41467-026-73323-6 (PMC13381710; doi:10.1038/s41467-026-73323-6)
Supplement: Supplementary file 1 — Supplementary Information [file 41467_2026_73323_MOESM1_ESM.pdf]

Supplementary Information for

Views of EU citizens on economic growth and implications for climate policy

Contents

Supplementary Figures S1–4

Supplementary Tables S1–13

Supplementary Discussion

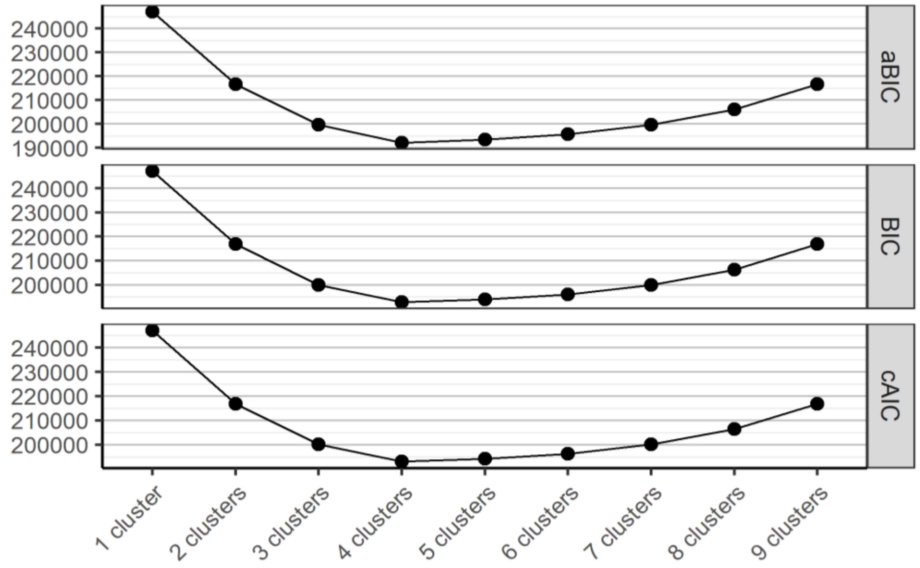

Fig. S1. Plot on the model fit for Latent Class Analysis (LCA) across different information criteria for 1- to 9-cluster solutions. aBIC stands for sample-size adjusted Bayesian Information Criterion, BIC for Bayesian Information Criterion, cAIC stands for consistent Akaike Information Criterion. Based on the results we chose 4 clusters ass this number consistently minimises the three information criteria. Total number of observations used n = 16,781.

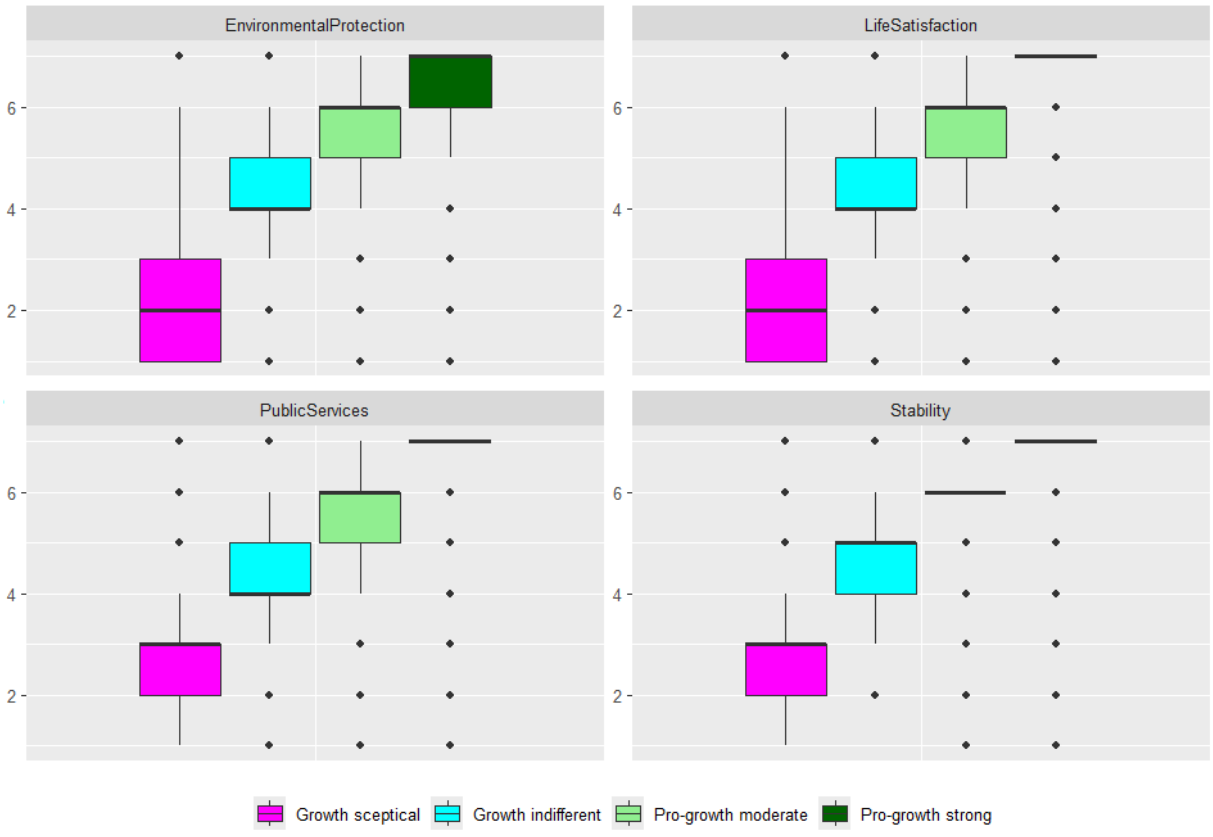

Fig. S2. Opinion clusters based on responses to the GEM statements on economic growth. Y-axis corresponds to respondents' answers on a 7-point Likert scale from "strongly disagree" (1) to "strongly agree" (7). Total number of observations used n = 16,781.

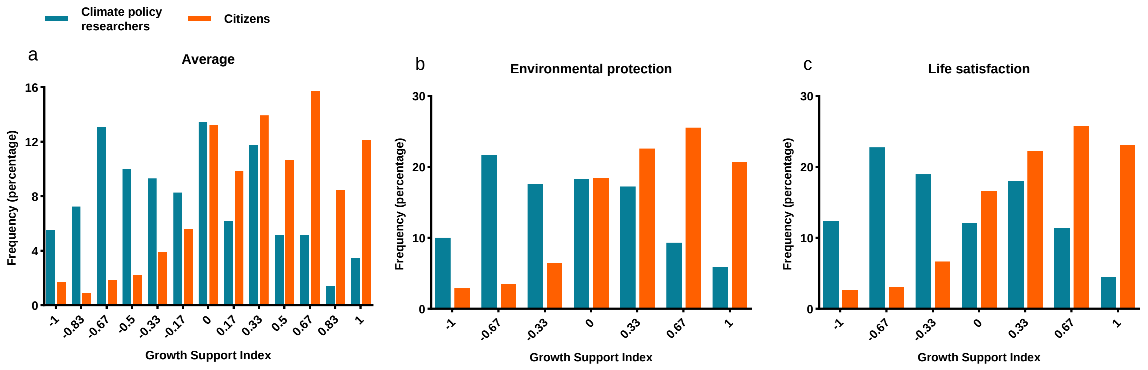

Fig. S3. Histograms of the Growth Support Index for climate policy researchers (blue) and citizens (orange). Panels show the distribution of responses using (a) the average of the two statements, (b) environmental protection, and (c) life satisfaction. Frequencies are normalised to percentages to allow direct comparison across the two groups. Total number of observations used n = 16,781.

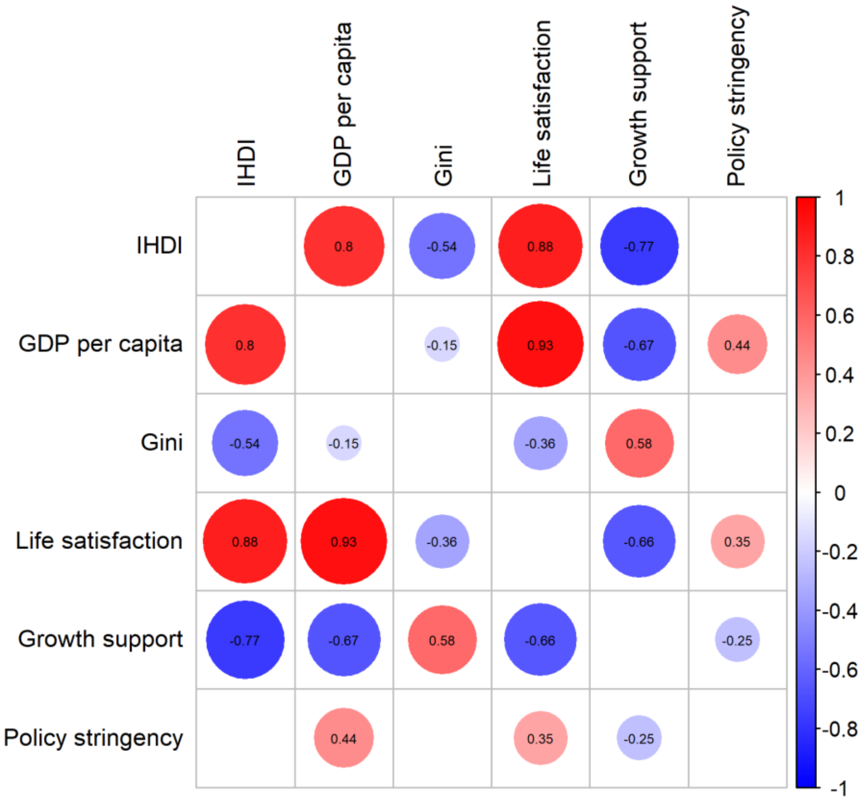

Fig S4. Correlation plot presenting Pearson correlations of variables presented in Figure 3 that are significant at the 5% level. Total number of observations used n = 13.

Table S1. OLS and Multiple Logit Regression Results. OLS results are reported in the second column, while the other three are for multiple logit. For multinomial logit, the pro-growth moderate cluster is used as a baseline. Standard errors are reported in parentheses. *p<0.1; **p<0.05; ***p<0.01.

	OLS	Multinomial model		
**Variable**	**Growth Support**	**Growth-sceptical**	**Growth-indifferent**	**Pro-growth (strong)**
**Sociodemographics**				
Gender	-0.009 (0.006)	-0.170*** (0.063)	0.192*** (0.042)	-0.044 (0.045)
Age	0.044*** (0.002)	-0.127*** (0.025)	-0.107*** (0.017)	0.165*** (0.018)
Education	0.018*** (0.005)	0.030 (0.048)	-0.087*** (0.032)	0.040 (0.033)
Household income	0.005*** (0.001)	-0.010 (0.012)	-0.019** (0.008)	0.018** (0.009)
Urban	0.002 (0.003)	0.027 (0.027)	-0.039** (0.018)	-0.018 (0.019)
Household size	-0.005** (0.003)	0.059** (0.026)	0.044** (0.018)	0.040** (0.020)
Car use	0.007*** (0.002)	-0.035* (0.021)	-0.028** (0.014)	-0.021 (0.016)
Flight frequency	-0.001 (0.001)	0.007 (0.005)	-0.014*** (0.005)	-0.010* (0.005)
**Political and climate views**				
Climate concern	-0.001 (0.004)	0.192*** (0.037)	0.071*** (0.026)	0.097*** (0.027)
Right-wing political orientation	0.016*** (0.001)	-0.045*** (0.014)	-0.042*** (0.009)	0.057*** (0.010)
Trust in national government	0.004* (0.002)	-0.110*** (0.026)	-0.012 (0.017)	-0.076*** (0.017)
Trust in EU government	0.023*** (0.002)	-0.128*** (0.026)	-0.072*** (0.017)	0.034** (0.017)
Climate policy knowledge	0.009*** (0.002)	-0.005 (0.020)	-0.028** (0.014)	0.055*** (0.014)
Support for climate policies	0.003 (0.003)	-0.107*** (0.030)	-0.050** (0.021)	-0.073*** (0.021)
Perceived climate action	0.026*** (0.002)	-0.152*** (0.023)	-0.023 (0.016)	0.075*** (0.017)
**Climate policy instruments**				
ETS support	-0.001 (0.003)	-0.074*** (0.027)	-0.031* (0.018)	-0.067*** (0.019)
Mandatory Insulation	0.013*** (0.002)	-0.132*** (0.020)	-0.029** (0.014)	0.036** (0.014)
Fossil Fuel Profits Tax	0.002 (0.002)	-0.001 (0.021)	-0.022 (0.014)	0.009 (0.014)
Intensive cattle farming ban	-0.012*** (0.002)	0.094*** (0.023)	0.031** (0.016)	-0.031** (0.016)
Private jet ban	0.002 (0.002)	-0.053** (0.023)	-0.034** (0.016)	-0.041** (0.016)
Advertising ban	-0.002 (0.002)	0.034 (0.025)	-0.020 (0.017)	0.021 (0.017)
ETS II for transport	0.005 (0.003)	-0.092*** (0.029)	0.014 (0.020)	-0.005 (0.021)
ETS II for heating	0.004 (0.003)	-0.002 (0.030)	0.004 (0.020)	0.008 (0.021)
ETS II for agriculture	0.001 (0.003)	0.001 (0.028)	-0.044** (0.019)	-0.019 (0.020)
CBAM support	-0.001 (0.002)	0.044* (0.024)	-0.022 (0.016)	-0.002 (0.016)
Beef Tax	-0.014*** (0.002)	0.032 (0.023)	0.087*** (0.015)	-0.034** (0.016)
Flight Tickets Tax	-0.015*** (0.002)	0.132*** (0.019)	0.061*** (0.013)	-0.010 (0.013)
EU rail fund	0.028*** (0.002)	-0.093*** (0.024)	-0.076*** (0.016)	0.077*** (0.018)
ICE car ban	-0.014*** (0.002)	0.086*** (0.021)	0.078*** (0.014)	0.012 (0.014)
**Values**				
Power (wealth)	-0.020 (0.028)	0.002 (0.019)	0.088*** (0.019)	-0.018 (0.020)
Universalism (equality)	0.001 (0.028)	-0.010 (0.019)	0.097*** (0.021)	0.022 (0.022)
Security (personal)	-0.082*** (0.028)	-0.036* (0.020)	0.129*** (0.022)	0.012 (0.023)
Security (national)	-0.165*** (0.027)	-0.156*** (0.019)	0.197*** (0.021)	0.048*** (0.022)
Hedonism	0.004 (0.027)	-0.065*** (0.018)	0.029 (0.019)	0.005 (0.019)
Self-direction	-0.122*** (0.028)	-0.111*** (0.020)	0.185*** (0.023)	0.035*** (0.024)
Benevolence	-0.004 (0.030)	-0.047** (0.020)	0.037* (0.022)	0.008** (0.023)
Achievement	-0.128*** (0.028)	-0.015 (0.019)	0.043** (0.019)	0.016*** (0.020)
Stimulation	-0.063** (0.027)	0.014 (0.018)	-0.077*** (0.018)	-0.005* (0.019)
Conformity	0.013*** (0.003)	-0.069*** (0.027)	0.008 (0.018)	0.022 (0.019)
Tradition	-0.133*** (0.024)	-0.040** (0.016)	0.025 (0.017)	0.014*** (0.018)
Universalism (nature)	-0.003 (0.003)	0.094*** (0.031)	-0.082*** (0.021)	-0.018 (0.023)
Constant	-0.940* (0.031)	3.918*** (0.326)	4.273*** (0.222)	-5.023*** (0.254)
Country Fixed Effects	Included		Included	
Observations	16,781		16,781	
R^2 / AIC	0.242		38,669.19	

Table S2. Survey questions and statements corresponding to sociodemographic, political views, and climate views variables.

Variable	Survey question/statement
**Sociodemographics**	
Gender	What is your gender?
Age	How old are you?
Education	What is the highest level of education that you have completed?
Household income	Which letter describes your household's total income, after tax and compulsory deductions, from all sources?
Urban	Which phrase best describes the area where you live?
Household size	Including yourself, how many people – including children – live regularly with you as members of this household?
Car use	How often do you use a car as a driver or a passenger?
Flight frequency	In total, how many flights did you take for private and personal travel in 2023 (not including business travel)?
**Political and climate views**	
Climate concern	How worried are you about climate change?
Right-wing political orientation	In politics, people sometimes talk of “left” and “right”. Where would you place yourself on this scale, where 1 means the left and 11 means the right?
Trust in national government	On a scale from 1 to 7, how much do you personally trust [country adjective] parliament?
Trust in EU government	On a scale from 1 to 7, how much do you personally trust the European parliament?
Perceived climate policy knowledge	Before today, how informed would you say you were about “Fit for 55” policies?
Personal support for climate policies	How much opposed, or in favour, are you of climate policies in general?
Perceived climate action	And how much opposed, or in favour, do you think an average politician in [country] is of climate policies in general?

Table S3. Interview statements relating to basic human values. Respondents were asked to think about how much each person is or is not like them on a six-point scale from "not at all like me" to "very much like me".

Motivational group	Value	Interview statement
Conservation	Conformity	It is important for this person to always behave properly. They want to avoid doing anything people would say is wrong.
	Security (national)	It is important to this person that the government ensures their safety against all threats. They want the state to be strong so it can defend its citizens.
	Security (personal)	It is important to this person to live in secure surroundings. They avoid anything that might endanger their safety.
	Tradition	Tradition is important to this person. They try to follow the customs handed down by their religion or their family.
Openness to change	Self-direction	It is important to this person to make their own decisions about what they do. They like to be free and not depend on others.
	Stimulation	This person looks for adventures and likes to take risks. They want to have an exciting life.
Self-enhancement	Achievement	Being very successful is important to this person. They hope people will recognize their achievements.
	Hedonism	Having a good time is important to this person. They like to "spoil" themselves.
	Power (wealth)	It is important to this person to be rich. They want to have a lot of money and expensive things.
Self-transcendence	Benevolence	It's very important to this person to help the people around them. They want to care for their well-being.
	Universalism (equality)	This person thinks it is important that every person in the world be treated equally. They believe everyone should have equal opportunities in life.
	Universalism (nature)	It is important to this person to respect nature. They want to be connected to nature.

Table S4. Survey descriptions of climate policy instruments. Respondents were asked how much they were opposed or in favour of the policies on a 7-point Likert scale from "strongly opposed" to "strongly in favour".

Variable	Survey description of policy instrument
ETS support	The EU-ETS is the world's largest carbon market. It sets limits on the amount of greenhouse gas emissions for various sectors of the economy, and forces companies to pay a price for their emissions. In turn, this leads to higher prices for consumers for high emission products, while also generating additional funds for governments across the EU.
ETS II for transport	A new ETS for fuels used for road transport? Road transport is the movement of people and goods on roads.
ETS II for heating	A new ETS for fuels used to heat buildings and homes?
ETS II for agriculture	A new ETS for agricultural production?
CBAM support	The EU will introduce a tax on imported goods that are made outside of the EU based upon how much greenhouse gases were emitted to produce them (Carbon Border Adjustment Mechanism).<br>If companies want to sell products made outside the EU, they must pay approximately the same fees for emitting greenhouse gases that the company would have had to pay if the products were made inside the EU.
Beef tax	An increase in taxes on beef so that the price of beef products doubles in [country].
Fossil fuel profits tax	An increase in the taxes paid by energy companies on profits earned from fossil fuels in [country].
Flight tickets tax	A tax on flights, increasing ticket prices by 50% in [country].
EU rail fund	The creation of an EU Rail Fund which may be used to expand the rail network and lower the cost of rail ticket prices within Europe by 50%.
Mandatory insulation	Mandatory insulation of residential buildings by 2040 to meet a minimum energy efficiency standard on the condition that the government pays at least half of the cost for low-income households in [country].
ICE car ban	The EU would ban the sale of all new petrol- and diesel-cars after 2035.
Intensive cattle farming ban	An EU-wide ban on intensive cattle (beef) farming by 2035.
Private jet ban	An EU-wide ban on private planes using fossil fuels departing from or arriving within the EU after 2035.
Advertising ban	An EU-wide ban on public advertisements of emissions-heavy products and services, such as flights and fossil fuel vehicles.

Table S5. Descriptive Statistics

Variable	Mean	Standard deviation	Min	Max
**Growth Support Index**	0.428	0.433	-1	1
Environmental protection	5.14	1.51	1	7
Lie Satisfaction	5.33	1.47	1	7
Public services	5.45	1.45	1	7
Stability	5.22	1.50	1	7
**Position in the growth-vs-environment**	2.77	0.919	1 (growth-sceptical)	4 (strong pro-growth)
**Sociodemographics**				
Gender	1.51	0.5	1	2
Age	3.20	1.41	1	5
Education	2.25	0.705	1	3
Household income	5.01	2.76	1	11
Urban	3.58	1.19	1	5
Household size	2.56	1.32	1	25
Car use	3.46	1.57	0	5
Flight frequency	1.44	5.06	0	100
**Political and climate views**				
Climate concern	3.29	1.07	1	5
Right-wing political orientation	6.25	2.42	1	11
Trust in national government	3.42	1.76	1	7
Trust in EU government	3.53	1.75	1	7
Climate policy knowledge	2.85	1.61	1	7
Support for climate policies	4.53	1.66	1	7
Perceived climate action	4.28	1.36	1	7
**Climate policy instruments**				
ETS support	3.98	1.59	1	7
Mandatory Insulation	4.51	1.82	1	7
Fossil Fuel Profits Tax	4.37	1.89	1	7
Intensive cattle farming ban	3.74	1.92	1	7
Private jet ban	4.71	1.82	1	7
Advertising ban	4.41	1.74	1	7
ETS II for transport	4.20	1.63	1	7
ETS II for heating	4.16	1.63	1	7
ETS II for agriculture	4.20	1.67	1	7
CBAM support	4.42	1.65	1	7
Beef Tax	2.96	1.86	1	7
Flight Tickets Tax	3.44	1.97	1	7
EU rail fund	5.25	1.57	1	7
ICE car ban	3.22	1.94	1	7
**Values**				
Power (wealth)	2.78	1.40	1	7
Universalism (equality)	4.55	1.29	1	7
Security (personal)	4.41	1.25	1	7
Security (national)	4.34	1.31	1	7
Hedonism	3.72	1.34	1	7
Self-direction	4.68	1.18	1	7
Benevolence	4.29	1.19	1	7
Achievement	3.24	1.41	1	7
Stimulation	3.08	1.42	1	7
Conformity	3.90	1.32	1	7
Tradition	3.68	1.48	1	7
Universalism (nature)	4.51	1.23	1	7

Table S6. Distribution of GS values across the four clusters

	Min	Median	Mean	Max
Growth-sceptical	-1.00	-0.42	-0.50	0.25
Growth-indifferent	-0.25	0.17	0.13	0.50
Pro-growth moderate	0.00	0.58	0.58	0.83
Pro-growth strong	-0.08	0.92	0.89	1.00

Table S7: Age and Gender Quotas : Population and Sample Composition by Age and Gender for Each Country

		Male					Female				
		18-29	30-39	40-49	50-59	60-74	18-29	30-39	40-49	50-59	60-74
Austria	Target (n)	159	153	143	169	174	150	148	143	170	191
Austria	Target (%)	9.9%	9.6%	8.9%	10.6%	10.9%	9.4%	9.3%	8.9%	10.6%	11.9%
Austria	Sample (n)	109	134	141	178	189	173	164	147	172	200
Czechia	Target (n)	136	152	186	143	185	128	142	176	140	212
Czechia	Target (%)	8.5%	9.5%	11.6%	8.9%	11.6%	8.0%	8.9%	11.0%	8.8%	13.3%
Czechia	Sample (n)	126	141	196	162	205	136	156	198	163	243
Denmark	Target (n)	181	140	142	156	184	173	135	142	154	193
Denmark	Target (%)	11.3%	8.8%	8.9%	9.8%	11.5%	10.8%	8.4%	8.9%	9.6%	12.1%
Denmark	Sample (n)	159	138	140	149	203	177	135	142	161	203
France	Target (n)	120	103	108	112	142	118	109	112	116	160
France	Target (%)	10.0%	8.6%	9.0%	9.3%	11.8%	9.8%	9.1%	9.3%	9.7%	13.3%
France	Sample (n)	93	98	115	122	167	100	106	114	132	186
Germany	Target (n)	152	149	134	175	194	140	141	133	173	209
Germany	Target (%)	9.5%	9.3%	8.4%	10.9%	12.1%	8.8%	8.8%	8.3%	10.8%	13.1%
Germany	Sample (n)	118	134	124	188	233	121	135	135	179	237
Greece	Target (n)	140	134	169	160	188	130	131	170	168	210
Greece	Target (%)	8.8%	8.4%	10.6%	10.0%	11.8%	8.1%	8.2%	10.6%	10.5%	13.1%
Greece	Sample (n)	129	130	236	196	115	141	161	242	187	66
Hungary	Target (n)	152	144	178	139	169	142	135	174	144	223
Hungary	Target (%)	9.5%	9.0%	11.1%	8.7%	10.6%	8.9%	8.4%	10.9%	9.0%	13.9%
Hungary	Sample (n)	128	147	183	137	172	140	143	167	160	223

		Male					Female				
		18-29	30-39	40-49	50-59	60-74	18-29	30-39	40-49	50-59	60-74
Italy	Target (n)	104	94	119	132	147	95	92	120	136	161
Italy	Target (%)	8.7%	7.8%	9.9%	11.0%	12.3%	7.9%	7.7%	10.0%	11.3%	13.4%
Italy	Sample (n)	97	97	124	139	153	85	97	122	141	167
Netherlands	Target (n)	130	106	100	120	145	126	103	101	120	149
Netherlands	Target (%)	10.8%	8.8%	8.3%	10.0%	12.1%	10.5%	8.6%	8.4%	10.0%	12.4%
Netherlands	Sample (n)	100	89	97	122	180	106	115	107	138	171
Poland	Target (n)	142	170	165	127	180	137	164	162	131	222
Poland	Target (%)	8.9%	10.6%	10.3%	7.9%	11.3%	8.6%	10.3%	10.1%	8.2%	13.9%
Poland	Sample (n)	137	163	155	129	182	147	166	172	135	221
Slovenia	Target (n)	104	114	130	119	149	92	101	117	115	159
Slovenia	Target (%)	8.7%	9.5%	10.8%	9.9%	12.4%	7.7%	8.4%	9.8%	9.6%	13.3%
Slovenia	Sample (n)	79	99	136	132	139	108	131	145	122	118
Spain	Target (n)	139	134	180	166	174	134	134	179	169	191
Spain	Target (%)	8.7%	8.4%	11.3%	10.4%	10.9%	8.4%	8.4%	11.2%	10.6%	11.9%
Spain	Sample (n)	130	125	178	166	189	129	135	176	163	199
Sweden	Target (n)	174	163	147	149	181	160	154	142	145	185
Sweden	Target (%)	10.9%	10.2%	9.2%	9.3%	11.3%	10.0%	9.6%	8.9%	9.1%	11.6%
Sweden	Sample (n)	141	163	135	160	199	136	153	148	158	204

Table S8: Education Quotas : Population and Sample Composition by Education Levels for Each Country

Country	Group	Low (%)	Middle (%)	High (%)
Austria	Target	14.0	49.0	37.0
Austria	Sample	1.6	51.0	47.4
Czechia	Target	42.0	37.0	21.0
Czechia	Sample	41.2	36.9	21.9
Germany	Target	17.0	50.0	33.0
Germany	Sample	15.7	50.8	33.6
Denmark	Target	17.8	39.3	42.9
Denmark	Sample	15.4	40.2	44.4
France	Target	16.0	41.0	42.0
France	Sample	19.0	27.7	53.4
Greece	Target	18.9	46.7	34.3
Greece	Sample	4.9	46.0	49.1
Hungary	Target	12.6	57.5	29.8
Hungary	Sample	11.6	55.6	32.8
Italy	Target	34.5	43.9	21.6
Italy	Sample	21.3	48.4	30.4
Netherlands	Target	19.3	36.4	44.3
Netherlands	Sample	20.2	36.5	43.4
Poland	Target	5.7	56.4	37.9
Poland	Sample	6.1	55.1	38.8
Slovenia	Target	11.5	54.7	33.8
Slovenia	Sample	4.1	55.8	40.2
Spain	Target	35.8	22.7	41.4
Spain	Sample	34.9	23.2	41.9
Sweden	Target	10.0	41.0	49.4
Sweden	Sample	7.6	42.3	50.1

Table S9: Survey Duration, Sample Size, and Attention Check Failure Rate by Country

Country	Median Duration (m:s)	Sample Size	Failed Attention Checks
Austria	20:18	1594	6.6%
Czechia	29:10	1726	6.3%
Germany	19:06	1597	6.4%
Denmark	20:46	1591	6.3%
Spain	22:56	1590	9.4%
France	21:19	1219	6.3%
Greece	20:43	1596	6.6%
Hungary	23:21	1593	7.7%
Italy	20:42	1210	3.3%
Netherlands	18:53	1219	6.5%
Poland	21:55	1595	6.1%
Sweden	23:40	1597	10.9%
Slovenia	22:59	1201	5.9%

Table S10. Lasso Regression Results. Standard errors are reported in parentheses. *p<0.1; **p<0.05; ***p<0.01.

Variable	Growth Support
**Sociodemographics**	
Gender	—
Age	0.045*** (0.002)
Education	0.014*** (0.004)
Household income	0.005*** (0.001)
Urban	—
Household size	—
Car use	0.007*** (0.002)
Flight frequency	—
**Political and climate views**	
Climate concern	—
Right-wing political orientation	0.015*** (0.001)
Trust in national government	0.004* (0.002)
Trust in EU government	0.024*** (0.002)
Climate policy knowledge	0.009*** (0.002)
Support for climate policies	—
Perceived climate action	—
**Climate policy instruments**	
ETS support	—
Mandatory Insulation	0.013*** (0.002)
Fossil Fuel Profits Tax	—
Intensive cattle farming ban	—
Private jet ban	—
Advertising ban	—
ETS II for transport	0.005* (0.003)
ETS II for heating	0.003 (0.003)
ETS II for agriculture	—
CBAM support	—
Beef Tax	–0.013*** (0.002)
Flight Tickets Tax	–0.015*** (0.002)
EU rail fund	0.029*** (0.002)
ICE car ban	–0.014*** (0.002)
**Values**	
Power (wealth)	0.013*** (0.003)
Universalism (equality)	0.005* (0.003)
Security (personal)	0.049*** (0.003)
Security (national)	0.024*** (0.003)
Hedonism	0.003 (0.002)
Self-direction	0.034*** (0.003)
Benevolence	0.005* (0.003)
Achievement	0.014*** (0.003)
Stimulation	—
Conformity	0.014*** (0.003)
Tradition	0.013*** (0.002)
Universalism (nature)	—
Constant	–0.893*** (0.025)
Country Fixed Effects	Included DK, ES, IT, NL and SL
Observations	16,781
R^2	0.239

Table S11. OLS Regression Results for Sociodemographics and Views. Standard errors are reported in parentheses. *p<0.1; **p<0.05; ***p<0.01.

Variable	Growth Support
**Sociodemographics**	
Gender	0.008 (0.006)
Age	0.050*** (0.002)
Education	0.014*** (0.005)
Household income	0.005*** (0.001)
Urban	0.007*** (0.003)
Household size	–0.006** (0.003)
Car use	0.016*** (0.002)
Flight frequency	–0.001* (0.001)
**Political and climate views**	
Climate concern	0.013*** (0.004)
Right-wing political orientation	0.025*** (0.001)
Trust in national government	0.004 (0.003)
Trust in EU government	0.020*** (0.003)
Climate policy knowledge	0.009*** (0.002)
Support for climate policies	0.005* (0.003)
Perceived climate action	0.042*** (0.002)
Constant	–0.427*** (0.029)
Country Fixed Effects	Included
Observations	16,781
R^2	0.105

Table S12. OLS Regression Results for Climate Policy Instruments. Standard errors are reported in parentheses. *p<0.1; **p<0.05; ***p<0.01.

Variable	Growth Support
**Climate policy instruments**	
ETS support	0.004 (0.003)
Mandatory Insulation	0.021*** (0.002)
Fossil Fuel Profits Tax	0.002 (0.002)
Intensive cattle farming ban	–0.021*** (0.002)
Private jet ban	–0.012*** (0.002)
Advertising ban	0.002 (0.002)
ETS II for transport	0.005* (0.003)
ETS II for heating	0.010*** (0.003)
ETS II for agriculture	0.004 (0.003)
CBAM support	0.008*** (0.002)
Beef Tax	–0.019*** (0.002)
Flight Tickets Tax	0.003 (0.002)
EU rail fund	0.045*** (0.002)
ICE car ban	–0.016*** (0.002)
Constant	0.103*** (0.016)
Country Fixed Effects	Included
Observations	18,372
R^2	0.082

Table S13. *OLS* Regression Results for Values. Standard errors are reported in parentheses. *p<0.1; **p<0.05; ***p<0.01.

Variable	Growth Support
**Values**	
Power (wealth)	0.007*** (0.003)
Universalism (equality)	0.005* (0.003)
Security (personal)	0.061*** (0.003)
Security (national)	0.025*** (0.003)
Hedonism	0.008*** (0.003)
Self-direction	0.049*** (0.003)
Benevolence	0.008*** (0.003)
Achievement	0.013*** (0.003)
Stimulation	–0.014*** (0.002)
Conformity	0.017*** (0.003)
Tradition	0.025*** (0.002)
Universalism (nature)	0.001 (0.003)
Constant	–0.521*** (0.021)
Country Fixed Effects	Included
Observations	18,372
R^2	0.165

Table S14. OLS Regression Results for Denmark, Germany and Spain. Standard errors are reported in parentheses. *p<0.1; **p<0.05; ***p<0.01.

	Multinomial model		
**Variable**	**Denmark**	**Germany**	**Spain**
**Sociodemographics**			
Gender	0.015 (0.022)	-0.013 (0.021)	0.036* (0.021)
Age	0.018** (0.009)	0.057*** (0.008)	0.023*** (0.009)
Education	0.003 (0.016)	0.035** (0.016)	0.005 (0.013)
Household income	0.011** (0.004)	0.009** (0.004)	-0.008* (0.005)
Urban	-0.002 (0.010)	0.006 (0.009)	0.024*** (0.009)
Household size	-0.016 (0.010)	-0.003 (0.010)	0.018** (0.009)
Car use	0.010 (0.008)	0.012* (0.007)	0.006 (0.007)
Flight frequency	-0.001 (0.002)	-0.0001 (0.002)	0.0005 (0.002)
**Political and climate views**			
Climate concern	0.0002 (0.015)	-0.008 (0.013)	-0.010 (0.013)
Right-wing political orientation	0.030*** (0.005)	0.021*** (0.006)	0.004 (0.004)
Trust in national government	0.015 (0.010)	0.013 (0.010)	0.002 (0.008)
Trust in EU government	0.004 (0.010)	0.021* (0.011)	0.021** (0.009)
Climate policy knowledge	0.017** (0.007)	0.010 (0.007)	-0.004 (0.006)
Support for climate policies	0.023** (0.011)	-0.034*** (0.010)	0.018* (0.010)
Perceived climate action	0.047*** (0.009)	0.037*** (0.008)	0.025*** (0.007)
**Climate policy instruments**			
ETS support	0.0005 (0.010)	-0.004 (0.010)	0.005 (0.008)
Mandatory Insulation	0.013* (0.007)	0.016** (0.007)	0.001 (0.007)
Fossil Fuel Profits Tax	0.001 (0.007)	-0.003 (0.007)	-0.005 (0.008)
Intensive cattle farming ban	-0.026*** (0.009)	-0.010 (0.008)	-0.018** (0.008)
Private jet ban	-0.004 (0.008)	0.010 (0.007)	-0.006 (0.009)
Advertising ban	0.003 (0.009)	-0.014* (0.008)	0.008 (0.009)
ETS II for transport	0.001 (0.011)	0.006 (0.010)	-0.022** (0.011)
ETS II for heating	0.007 (0.010)	0.008 (0.010)	0.016 (0.011)
ETS II for agriculture	-0.005 (0.009)	0.006 (0.010)	-0.012 (0.010)
CBAM support	0.022*** (0.008)	-0.006 (0.008)	0.007 (0.008)
Beef Tax	-0.014 (0.008)	-0.016** (0.007)	-0.012 (0.008)
Flight Tickets Tax	-0.009 (0.007)	-0.015** (0.006)	-0.008 (0.007)
EU rail fund	0.010 (0.008)	0.040*** (0.008)	0.035*** (0.009)
ICE car ban	-0.003 (0.007)	-0.015** (0.007)	-0.005 (0.007)
**Values**			
Power (wealth)	-0.004 (0.010)	0.011 (0.009)	0.009 (0.009)
Universalism (equality)	-0.020** (0.009)	0.001 (0.009)	-0.002 (0.011)
Security (personal)	0.032*** (0.009)	0.030*** (0.010)	0.028** (0.011)
Security (national)	0.033*** (0.009)	0.043*** (0.009)	0.051*** (0.010)
Hedonism	0.011 (0.010)	0.011 (0.009)	0.018* (0.009)
Self-direction	0.029*** (0.009)	0.038*** (0.010)	0.051*** (0.010)
Benevolence	0.023** (0.010)	0.005 (0.010)	-0.006 (0.011)
Achievement	0.019* (0.010)	0.034*** (0.009)	0.005 (0.010)
Stimulation	0.005 (0.009)	-0.015* (0.009)	0.013 (0.009)
Conformity	0.020** (0.009)	0.010 (0.009)	0.035*** (0.009)
Tradition	0.006 (0.008)	0.026*** (0.008)	0.007 (0.008)
Universalism (nature)	-0.020** (0.010)	0.010 (0.010)	0.002 (0.012)
Constant	-1.015*** (0.106)	-1.092*** (0.099)	-0.904*** (0.099)
Observations	1,327	1,436	1,359
R^2	0.263	0.335	0.273

Supplementary Discussion

Comparison of growth positions between European citizens and climate policy researchers

While in the main text of the paper we examined how various factors shape citizens' perceptions of economic growth, an important question remains: how do these views compare to those of experts who influence climate policy? Understanding discrepancies between public opinion and expert perspectives is relevant as these can affect the feasibility and acceptance of sustainability policies. The four statements used to elicit preferences towards economic growth were originally developed as the GEM survey module[28]. Its items were validated for both the general public and experts[27] (see Methods for more details).

This survey instrument was previously used in a 2021 global survey of 789 climate policy researchers, 209 of whom were based in the same 13 EU countries covered in the public survey at the time of the survey[16]. This provides a useful reference for comparing the views of European citizens and policy researchers. Panel A of Figure S5 maps the distribution of responses from citizens and researchers to the GEM items after separate Latent Class Analysis. This identified the optimum number of clusters for policy researchers as three, namely pro-growth, growth-indifferent and growth-sceptical views. In contrast, the optimal clustering for citizens is fourfold, also differentiating between strong and moderate pro-growth attitudes.

Panel B of Figure S5 compares the distributions of these clusters among citizens and researchers. European citizens demonstrate considerably more pro-growth tendencies than climate policy researchers, with 60% aligning with pro-growth views (moderate and strong) compared to only 14% of researchers. Growth-scepticism is comparatively less prevalent among the public, with only 8% aligning with it compared to 35% of academic researchers, while growth-indifference is also lower among citizens (32%) than among researchers (51%). The substantial gap in scepticism towards economic growth between citizens and researchers is consistent with findings from previous studies for Australia, the United Kingdom and Spain[25,26].

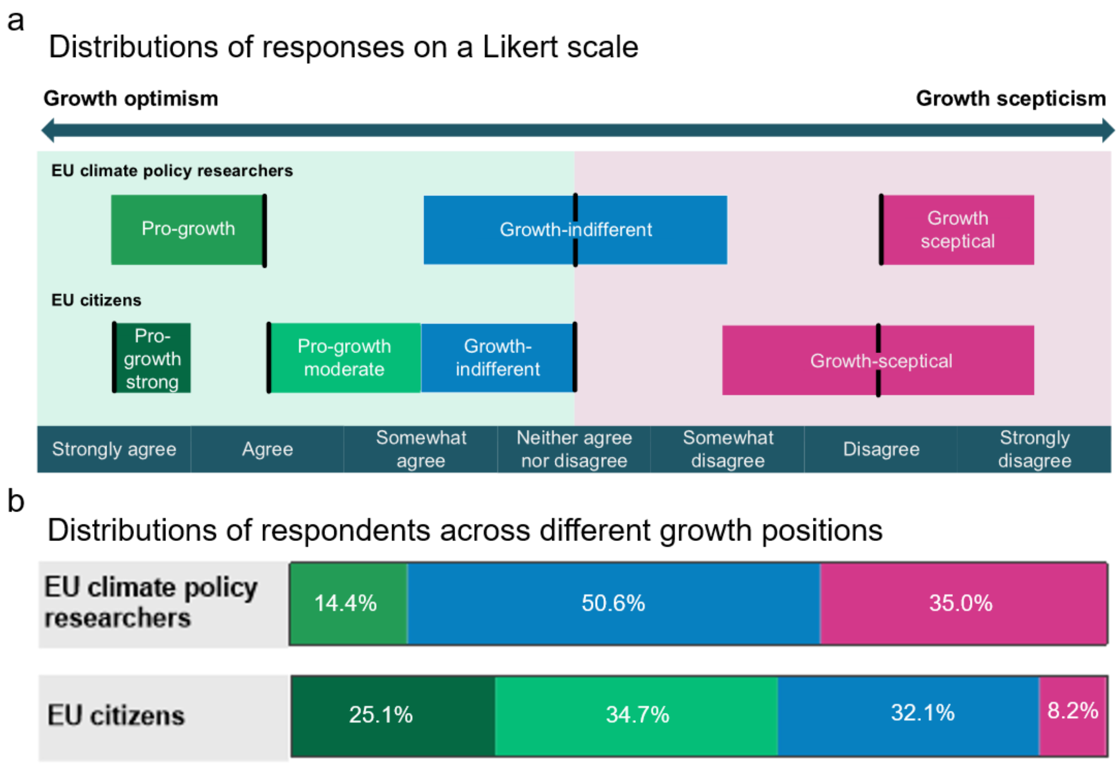

Fig. S5. Growth positions of EU citizens compared to climate policy researchers. (a) stylistic comparison of the distributions of respondents to expert and citizen surveys using two identical GEM statements on environmental protection and life satisfaction. Coloured boxes represent the interquartile ranges, and black lines represent the median survey responses of each cluster. **(b)** Distributions of growth positions from the present study for EU citizens (n=16,781) and from King et al. (2023) [10] for EU climate policy researchers, selecting for researchers resident in the 13 countries covered by the citizen survey (n=209). Labels of growth positions correspond to those with the same colours in Panel A.

Note that the purpose of Figure S5 is not to provide a statistical comparison in terms of mean or variance but to illustrate the concentration of the population of citizens and experts on distinct parts of the Likert scale. Figure S3 in the Supplementary Information further demonstrates this point through histograms of responses rescaled to the -1 to +1 Growth Support Index. When interpreting the differences between the public and the academics, it is also important to keep in mind that factors beyond knowledge of climate policy may partly explain the observed divergence. Climate policy researchers likely lean towards certain sociodemographic characteristics, and there is also a three-year gap between the surveys. During this period, events such as the Ukraine war and associated energy price rises have become pressing concerns in Europe. Issues related to security, migration and inflation may therefore contribute to the greater support for economic growth seen in the citizen survey [37,38].
