# Supplementary material for: Views of EU citizens on economic growth and implications for climate policy
Source: Nat Commun. 2026 May 19;17:6580. doi: 10.1038/s41467-026-73323-6 (PMC13381710; doi:10.1038/s41467-026-73323-6)
Supplement: Supplementary file 2 — Reporting Summary [file 41467_2026_73323_MOESM2_ESM.pdf]

nature portfolio

Corresponding author(s): Ivan Savin

Last updated by author(s): Mar 27, 2026

Reporting Summary

Nature Portfolio wishes to improve the reproducibility of the work that we publish. This form provides structure for consistency and transparency in reporting. For further information on Nature Portfolio policies, see our Editorial Policies and the Editorial Policy Checklist.

Statistics

For all statistical analyses, confirm that the following items are present in the figure legend, table legend, main text, or Methods section.

n/a	Confirmed	
☐	☒	The exact sample size (*n*) for each experimental group/condition, given as a discrete number and unit of measurement
☐	☒	A statement on whether measurements were taken from distinct samples or whether the same sample was measured repeatedly
☒	☐	The statistical test(s) used AND whether they are one- or two-sided<br>*Only common tests should be described solely by name; describe more complex techniques in the Methods section.*
☐	☒	A description of all covariates tested
☒	☐	A description of any assumptions or corrections, such as tests of normality and adjustment for multiple comparisons
☐	☒	A full description of the statistical parameters including central tendency (e.g. means) or other basic estimates (e.g. regression coefficient) AND variation (e.g. standard deviation) or associated estimates of uncertainty (e.g. confidence intervals)
☐	☒	For null hypothesis testing, the test statistic (e.g. *F*, *t*, *r*) with confidence intervals, effect sizes, degrees of freedom and *P* value noted<br>*Give P values as exact values whenever suitable.*
☒	☐	For Bayesian analysis, information on the choice of priors and Markov chain Monte Carlo settings
☒	☐	For hierarchical and complex designs, identification of the appropriate level for tests and full reporting of outcomes
☒	☐	Estimates of effect sizes (e.g. Cohen's *d*, Pearson's *r*), indicating how they were calculated

Our web collection on statistics for biologists contains articles on many of the points above.

Software and code

Policy information about availability of computer code

Data collection	The data collection and research project were pre-registered at OSF (https://osf.io/9dmvh/).
Data analysis	The replication data and code are available from GitHub at https://doi.org/10.5281/zenodo.19666333. We used R version 4.4.1

For manuscripts utilizing custom algorithms or software that are central to the research but not yet described in published literature, software must be made available to editors and reviewers. We strongly encourage code deposition in a community repository (e.g. GitHub). See the Nature Portfolio guidelines for submitting code & software for further information.

Data

Policy information about availability of data

All manuscripts must include a data availability statement. This statement should provide the following information, where applicable:

- Accession codes, unique identifiers, or web links for publicly available datasets
- A description of any restrictions on data availability
- For clinical datasets or third party data, please ensure that the statement adheres to our policy

he replication data and code are available from GitHub at https://doi.org/10.5281/zenodo.19666333

Research involving human participants, their data, or biological material

Policy information about studies with human participants or human data. See also policy information about sex, gender (identity/presentation), and sexual orientation and race, ethnicity and racism.

Reporting on sex and gender	All respondents were over 18 years of age at the time of the survey, and we adopted quota-based sampling methodology for demographic representivity: age and gender interlocked, and education see (Tables S7 and S8 in Supplementary Information for comparison of sample with population).
Reporting on race, ethnicity, or other socially relevant groupings	We do not report race, or ethnic characteristics.
Population characteristics	See behavioural & social sciences study design
Recruitment	We conducted a survey to collect data from 19,328 validated respondents in Austria, Czech Republic, Denmark, France, Germany, Greece, Italy, Hungary, Netherlands, Poland, Slovenia, Spain and Sweden from June 24 to August 27, 2024. We recruited respondents drawing upon an online panel provided by Dynata. All respondents were over 18 years of age at the time of the survey. The survey instrument was fielded in the main official languages for each country. The original survey was written in English, professionally translated into these languages, and then were reviewed by native speakers to assure accuracy.
Ethics oversight	The survey instrument and data collection were approved by the ETH-Zurich Ethics Committee (EK 2024-N-141), and was pre-registered at OSF (https://osf.io/9dmvh/).

Note that full information on the approval of the study protocol must also be provided in the manuscript.

Field-specific reporting

Please select the one below that is the best fit for your research. If you are not sure, read the appropriate sections before making your selection.

☐ Life sciences ☒ Behavioural & social sciences ☐ Ecological, evolutionary & environmental sciences

For a reference copy of the document with all sections, see nature.com/documents/nr-reporting-summary-flat.pdf

Behavioural & social sciences study design

All studies must disclose on these points even when the disclosure is negative.

Study description	Study is a quantitative design using original survey data collected in June 24 to August 27, 2024 from 13 countries in the EU. Full analytical procedures presented in the findings are described in the Methods.
Research sample	Sample was collected in partnership with a commercial panel provider, Dynata. All respondents were over 18 years of age at the time of the survey, and we adopted quota-based sampling methodology for demographic representativity: age and gender interlocked, and education see (Tables S7 and S8 in Supplementary Information for comparison of sample with population). Therefore, the data is representative. The final sample size after excluding all missing observations is 16,781 respondents
Sampling strategy	Data was collected using an online based survey. The survey was programmed using Qualtrics software.
Data collection	Data was collected using an online based survey. The survey was programmed using Qualtrics software. The respondent was blind to any experimental conditions.<br>Approval for the study and survey was granted by the ETH Zurich Ethics Committee (EK 2024-N-141). All participants signed an informed consent.
Timing	We conducted a survey to collect data from 19,328 validated respondents in Austria, Czech Republic, Denmark, France, Germany, Greece, Italy, Hungary, Netherlands, Poland, Slovenia, Spain and Sweden from June 24 to August 27, 2024.
Data exclusions	We adopted attention checks-based methods to flag low-quality responses with the following criteria : response duration below 45% country-specific median, incorrect response to item asking respondents to select 'other' in a single-display question, incorrect response to asking respondents to select 'somewhat like me' within a matrix of questions. If respondents failed 2 out of the 3 responses, they were replaced in the sampling process and excluded from the final analytical sample (6.3%.). The country specific median duration, final analytical sample size and % of sample that failed attention checks is displayed in Table S9 in Supplementary Information.
Non-participation	Outside of the respondents that were excluded from the analytic sample (described in the section above), 3.8% of respondents invited to participate did not provide their informed consent to participate in the study, and were therefore not included in the study (and no data was collected from them). Further, respondents who started but did not fully complete the questionnaire are excluded from the final analytical sample.
Randomization	N/A

Reporting for specific materials, systems and methods

We require information from authors about some types of materials, experimental systems and methods used in many studies. Here, indicate whether each material, system or method listed is relevant to your study. If you are not sure if a list item applies to your research, read the appropriate section before selecting a response.

Materials & experimental systems

n/a	Involved in the study
☒	☐ Antibodies
☒	☐ Eukaryotic cell lines
☒	☐ Palaeontology and archaeology
☒	☐ Animals and other organisms
☒	☐ Clinical data
☒	☐ Dual use research of concern
☒	☐ Plants

Methods

n/a	Involved in the study
☒	☐ ChIP-seq
☒	☐ Flow cytometry
☒	☐ MRI-based neuroimaging

Plants

Seed stocks	*Report on the source of all seed stocks or other plant material used. If applicable, state the seed stock centre and catalogue number. If plant specimens were collected from the field, describe the collection location, date and sampling procedures.*
Novel plant genotypes	*Describe the methods by which all novel plant genotypes were produced. This includes those generated by transgenic approaches, gene editing, chemical/radiation-based mutagenesis and hybridization. For transgenic lines, describe the transformation method, the number of independent lines analyzed and the generation upon which experiments were performed. For gene-edited lines, describe the editor used, the endogenous sequence targeted for editing, the targeting guide RNA sequence (if applicable) and how the editor was applied.*
Authentication	*Describe any authentication procedures for each seed stock used or novel genotype generated. Describe any experiments used to assess the effect of a mutation and, where applicable, how potential secondary effects (e.g. second site T-DNA insertions, mosiacism, off-target gene editing) were examined.*
