# Supplementary material for: Views of EU citizens on economic growth and implications for climate policy
Source: Nat Commun. 2026 May 19;17:6580. doi: 10.1038/s41467-026-73323-6 (PMC13381710; doi:10.1038/s41467-026-73323-6)
Supplement: Supplementary file 3 — Transparent Peer Review file [file 41467_2026_73323_MOESM3_ESM.pdf]

nature portfolio

Peer Review File

Views of EU citizens on economic growth and implications for climate policy

Corresponding Author: Dr Ivan Savin

This file contains all reviewer reports in order by version, followed by all author rebuttals in order by version.

Version 0:

Reviewer comments:

Reviewer #1

(Remarks to the Author)
This is an interesting paper that addresses an essential question of political behaviour. Understanding public opinion of green growth attitudes relative to the perspective of experts will be essential for the success of climate policy efforts. The questions set out in the paper could provide a major contribution to this understanding. However, there are some major methodological concerns that need to be addressed before publication.

1. Measurement incompatibility
The comparison of public opinion with expert data as it is done in this paper is problematic. First, public opinion will depend much more on how the topic is framed and what is salient at the moment, therefore it will be difficult to compare the more volatile public position to the much more informed and stable experts position based on a single time period. Just using the one point in time for public opinion compounds this issue. Additionally, different clustering makes comparison very difficult: researchers were classified into 3 clusters (green growth, agrowth, and degrowth) while citizens were classified into 4 clusters (strong pro-growth, moderate pro-growth, agrowth, and degrowth). Item Response Theory (IRT) or Confirmatory Factor Analysis (CFA) could potentially allow a better comparison by estimating a continuous scale. This could be possible since both surveys shared two items, they could anchor the latent trait model.
2. Sampling strategy
The authors should provide more detailled information on the sampling strategies and data generating process. The authors mention the collected data for each country is representative in terms of age, gender and education. There is no mention of sampling method (random, stratified, quota, etc.), no mention of online vs. offline collection, no mention of response rates, no sampling weights discussed, and no comparison to population parameters shown. Similarly, insufficient information on the researcher sample: how researchers were sampled, what constitutes a "climate policy researcher," response rates, and representativeness of the researcher sample.
3. Modelling approach
the authors provide no clear justification for the selection of variables. The model in its current form includes 53 (!!) covariates (including the country fixed effects), and it likely suffers from overfitting. I see some crucial misalignments between the multinomial model and the OLS: while the authors state that education leads to higher growth support, this is not actually the case when looking at the multinomial model where only the probability of an agrowth position is affected. Much more care is necessary to correctly interpret the findings of multinomial models Furthermore, there are several instances where we can observe significant effects in the multinomial while the OLS does not show significant effects like for gender and urban population.
This observation makes me wonder about the role of some sociodemographic features such as urban/rural differences and education. I would expect that researchers by default live in urban regions and are highly educated; according to the authors' model both of these features increase the probability of agrowth positions. It is therefore possible that the differences between researchers and the general public are also driven by demographic differences. It would be more interesting to compare researchers with more comparable demographics.
4. Limited Causal Links and Theoretical Scope
In its current form, the analysis is very descriptive in nature. I am convinced the data at hand could be leveraged for clearer causal identification strategy that would help to form clearer policy recommendations

(Remarks on code availability)

Reviewer #2

(Remarks to the Author)
I co-reviewed this manuscript with one of the reviewers who provided the listed reports. This is part of the Nature Communications initiative to facilitate training in peer review and to provide appropriate recognition for Early Career Researchers who co-review manuscripts.

(Remarks on code availability)

Reviewer #3

(Remarks to the Author)
This paper offers a valuable empirical contribution by examining public attitudes toward economic growth and their alignment with climate policy preferences across 13 EU countries. Based on a large, representative survey and robust analytical techniques (notably Latent Class Analysis and regression modeling), it provides a nuanced typology of growth attitudes—distinguishing between strong/moderate pro-growth, agrowth, and degrowth orientations—and compares these with expert opinion. The data are rich, the methodology is transparent, and the results are potentially highly relevant to ongoing debates in sustainability and political economy.

That said, several areas require further development to meet the standards of a journal such as NC. Most notably, the conceptual framing of “growth” remains under-theorized. The categories of green growth, agrowth, and degrowth are introduced descriptively, but the paper does not engage deeply with the normative, political, or epistemological tensions underlying these positions. This limits the interpretive depth of the findings, especially where the authors highlight surprising correlations—such as the positive association between pro-growth views and values like benevolence and equality—without probing whether respondents interpret “growth” in instrumental, moral, or nationalistic terms.

In addition, the analysis at times blurs the line between correlation and causation. For example, the claim that political trust leads to stronger growth support is not supported by the cross-sectional design and should be more cautiously stated. The role of gender, climate concern, and lifestyle proxies (e.g., flight frequency) in shaping growth attitudes is reported but insufficiently theorized. These findings could benefit from engagement with existing literature on gendered environmental attitudes or psychological mechanisms like motivated reasoning.

Finally, while the comparison to expert surveys is a useful framing device, the implications of the public–academic gap are underdeveloped. What does this misalignment suggest about the prospects for democratic legitimacy or effective climate governance? The discussion gestures at policy implications but remains general.

In sum, the paper is empirically strong and timely, but would benefit from tighter conceptual framing, more careful interpretation of associations, and deeper engagement with the theoretical stakes of its findings. A revised version with these improvements would be a strong candidate for publication.

(Remarks on code availability)
The link led to a GitHub site "404 not available"

Reviewer #4

(Remarks to the Author)
The paper explores public attitudes towards (green) growth and post-growth in light of climate change. This is certainly an important topic to study and such the paper could make an interesting contribution to the literature. The main value-added / novelty is the rich 19,000 responses-strong survey in 13 EU countries. The statistical manipulations to analyze those data are relatively straightforward.

I have the following observation to strengthen the manuscript before it is ready for publication.

1. Definitions of growth paradigms. Unlike other papers, this survey did not ask people directly about their views on different notions of growth. Instead, attitudes are derived from the answers to four proxy questions. I'm not sure the proxy answers map neatly to the usual growth paradigms found in the literature. For example, disagreeing with the statement "economic growth is necessary to finance environmental protection" is not the same as agreeing with the statement "economic growth is incompatible with environmental protection" and only the latter is an unequivocal degrowth position. Perhaps I disagree with the statement because I think we can already afford environmental protection, without having a view on (de)growth. Similarly, the statements about public services and stability are statements about conventional economic growth, rather than green growth (and indeed the green growth label is not consistently used). Or, as a final example, respondents who are neutral to the four statements may register a "don't know" answer or subscribe to notions of weak green growth ("we can decouple economic activity from environmental damage"; Jacobs 2013) rather than being growth-agnostic. The authors should review the labels they assign to the various positions.

2. Statistical model. I am not a statistician, but was wondering about the inclusion of of the policy instruments variables in the

main regressions. These strike me as endogenous / correlated with other covariates. For example, if I had to explain ETS support, I would include variables like climate concern, trust and the socio-demographic variables. This might then suggest eg a two-stage least square approach. A corollary of this technical point is that the correlations shown in Figure 6 may simply reflect the fact that both growth attitudes and policy preferences are shaped by the same socio-economic factors.

3. Researcher vs public attitudes. The comparison of public attitudes to those of researchers is interesting, but a few caveats may be required. Even if the questions were exactly the same (which I assume to be the case), the authors are comparing a 2021 researcher survey with a 2024 public survey. Difference may be due either to the different respondents or the different timeframe. Most notably, the Ukraine war from 2022 onward and associated energy price hikes / economic fragility may well have affected growth attitudes.

4. A couple of smaller points

- p.10: why is the lack of correlation with climate concerns unexpected? It is perfectly consistent with green growth views (even if these are not captured very well by the four proxy questions see above).

p.15: why is urban residence a proxy for a high-carbon lifestyle? City dwellers are more likely to use public transport for example. They may (or may not) be better off, but you control for income.

- p.18: it is obviously fine to rescale the ordinal responses from the original Likert scale. By the same token, it is unclear what is being gained from it. A 1-7 scale is as intuitive as a -1 to 1 one.

- Chart S1 in the Supplementary Material should be made self-explanatory. What has been analysed here (LCA presumably), what is the scale of the y axis, what do the various acronyms (cAIC etc) stand for.

(Remarks on code availability)

Version 1:

Reviewer comments:

Reviewer #4

(Remarks to the Author)
I continue to believe that this is an interesting empirical paper that sheds important light on public attitudes about growth in Europe.

The authors have responded thoughtfully and comprehensively to reviewer comments. Mostly this was achieved by changes to interpretations (e.g. emphasis on correlation vs causality) and framing (e.g. the growth labels) and by adding further explanations (e.g. on sampling strategy), rather than undertaking different analysis, although there are added statistical tests (e.g. LASSO).

I still believe the paper would be stronger without the section that compares the public survey with the researcher survey results. This comparison is dubious for the reasons set out by this and other reviewers before. These are now set out, but it begs the question why the paper contains a comparison that is explicitly acknowledged to be problematic.

(Remarks on code availability)

Reviewer #5

(Remarks to the Author)
This manuscript analyzes public opinion about economic growth using original data collected in 13 European countries, complemented with a survey of experts in multiple European countries. I agree with the authors’ argument that public (and experts’) opinions about the relation between environmental protection and economic growth is often more complex than a simple trade-off between the two and I also appreciate that they have invested a lot of efforts in terms of data collection and analysis. However, even after the revision the paper continues to have several major weaknesses due to which I am unable to recommend it for publication.
First, the paper still lacks strong theoretical framework. The post-growth paradigm is discussed rather superficially. There is also no discussion of how socio-demographics, human values, trust, political ideology, specific policy preferences, etc. are expected to correlate with their dependent variable. While I acknowledge that the paper is exploratory and thus does not necessarily need to have testable hypotheses, there needs to be a justification why those independent variables might matter; otherwise, the reader gets an impression that the authors simply used any variable they had at hand. This also makes it difficult to interpret the results, many of which are quite surprising – e.g. more educated and universalist people being more likely to support growth, weak correlation between growth skepticism and environmental concern or environmental policy support, etc. – and post-hoc explanations/speculations are not particularly convincing.
Second, the authors cannot seem to make up their mind whether their dependent variable is continuous or nominal. Based

on their argument about the complexity of opinions about growth vs. environment, and also the literature they cite (e.g. several papers by Van Den Bergh, Savin and Drews), segmentation approach seems more appropriate, so personally I would opt for clustering, but if they prefer a continuum, they should justify why. In any case, the two approaches are incompatible and cannot be used simultaneously, even for robustness test.
Third, related to the previous point, the fact that the authors identify four clusters (as opposed to three clusters identified by Van Den Bergh and Drews) is problematic as ‘pro-growth strong’ and ‘pro-growth moderate’ clusters both correspond to a single ‘green growth’ position. I acknowledge that the choice of four clusters is statistically correct (judged by lowest cAIC and BIC), but substantively it makes more sense to treat them as a single group as both support growth, even if with different intensity.
Fourth, given that the data is available for 13 countries only, which is too few for multi-level modelling, I do not think that the substantive claims can be made about the effects of contextual variables, such as affluence and inequality. Reporting correlations with 13 cases should definitely be avoided. Instead, I would suggest using country fixed-effects models and then also replicating pooled data results at country level for a robustness test to make at least some use of cross-national nature of the data (in addition to reporting country-level distribution of clusters in Figure 1 which is completely blurry in the pdf).

(Remarks on code availability)

Version 2:

Reviewer comments:

Reviewer #4

(Remarks to the Author)
I have reviewed the revised manuscript and believe that it is ready for publication.

Over multiple revisions the authors have responded satisfactorily to my requests. The framing seems fine now and results are described as correlations, rather than causal inference.The, in my opinion, questionable comparison between expert and public opinion has been moved to the supplementary material, which is a good solution.

I also feel the authors have responded adequately to the second reviewer. In particular I concur that working with four clusters makes sense since this is driven by the data. I am comfortable with the parallel discussion of both continuous and clustered results.

Congratulations on an interesting study.

(Remarks on code availability)

Open Access This Peer Review File is licensed under a Creative Commons Attribution 4.0 International License, which permits use, sharing, adaptation, distribution and reproduction in any medium or format, as long as you give appropriate credit to the original author(s) and the source, provide a link to the Creative Commons license, and indicate if changes were made.
In cases where reviewers are anonymous, credit should be given to 'Anonymous Referee' and the source.
The images or other third party material in this Peer Review File are included in the article's Creative Commons license, unless indicated otherwise in a credit line to the material. If material is not included in the article's Creative Commons license and your intended use is not permitted by statutory regulation or exceeds the permitted use, you will need to obtain permission directly from the copyright holder.
To view a copy of this license, visit https://creativecommons.org/licenses/by/4.0/

Response to reviewers

Manuscript Number: NCOMMS-25-33850

Views of EU citizens on economic growth and implications for climate policy

Response to reviewer #1

(Our response is in Italics)

This is an interesting paper that addresses an essential question of political behaviour. Understanding public opinion of green growth attitudes relative to the perspective of experts will be essential for the success of climate policy efforts. The questions set out in the paper could provide a major contribution to this understanding. However, there are some major methodological concerns that need to be addressed before publication.

Thank you for your positive evaluation and suggestions to improve the quality of our paper.

1. Measurement incompatibility

The comparison of public opinion with expert data as it is done in this paper is problematic. First, public opinion will depend much more on how the topic is framed and what is salient at the moment, therefore it will be difficult to compare the more volatile public position to the much more informed and stable experts position based on a single time period. Just using the one point in time for public opinion compounds this issue. Additionally, different clustering makes comparison very difficult: researchers were classified into 3 clusters (green growth, agrowth, and degrowth) while citizens were classified into 4 clusters (strong pro-growth, moderate pro-growth, agrowth, and degrowth). Item Response Theory (IRT) or Confirmatory Factor Analysis (CFA) could potentially allow a better comparison by estimating a continuous scale. This could be possible since both surveys shared two items, they could anchor the latent trait model.

Thank you for this comment. The purpose of Figure 1 is not to make an exact statistical comparison (such as variance etc) but to illustrate the concentration of the population in different ranges of the Likert scale with most of general public being pro-growth and many experts being growth sceptical. This is why we call it "stylized comparison" in the manuscript. To further emphasize this, we add a note in the revised manuscript and an additional figure in the Supplementary Information comparing distributions of the two samples.
We also fully agree with the reviewer that the framing (with experts being more informed) but also the timing of the surveys (2021 for policy experts and 2024 for general public) make the comparison difficult since events like COVID-19 and the Ukraine war may have affected the responses. We now discuss these limitations when presenting the comparison of views with climate policy researchers in the revised manuscript.

Finally, we prefer not to use IRT/CFA as these are instruments to validate new survey items but the items we use have been earlier used by Drews et al. (2019) who have shown that the clustering results produced based on these items are strongly correlated with responses on the direct question regarding preferred position in the growth-vs-environment debate both for general public and for the experts. Incidentally we would like to point out that Drews et al. (2019) compared a population of 814 international experts from 2015 and a representative sample of

1004 individuals in Spain surveyed in 2014 using exactly the same questions and framing on growth vs environment debate and finding similar cluster distribution across the two samples.

2. Sampling strategy

The authors should provide more detailed information on the sampling strategies and data generating process. The authors mention the collected data for each country is representative in terms of age, gender and education. There is no mention of sampling method (random, stratified, quota, etc.), no mention of online vs. offline collection, no mention of response rates, no sampling weights discussed, and no comparison to population parameters shown. Similarly, insufficient information on the researcher sample: how researchers were sampled, what constitutes a "climate policy researcher," response rates, and representativeness of the researcher sample.

The novel contribution of our paper is the survey of the general public, which we now explicitly mention in the revised manuscript. The results of the climate policy researchers come from a different paper that has already been published (King et al. 2023) and are only used in our paper for comparative purposes. We have added detailed information on data collection and its representativeness of the general public in the 13 EU countries in the Methods section as well as the Supplementary Information. Detailed information on data collection of the sample of climate policy researchers is contained in King et al. (2023).

3. Modelling approach

the authors provide no clear justification for the selection of variables. The model in its current form includes 53 (!!) covariates (including the country fixed effects), and it likely suffers from overfitting. I see some crucial misalignments between the multinomial model and the OLS: while the authors state that education leads to higher growth support, this is not actually the case when looking at the multinomial model where only the probability of an agrowth position is affected. Much more care is necessary to correctly interpret the findings of multinomial models Furthermore, there are several instances where we can observe significant effects in the multinomial while the OLS does not show significant effects like for gender and urban population.
This observation makes me wonder about the role of some sociodemographic features such as urban/rural differences and education. I would expect that researchers by default live in urban regions and are highly educated; according to the authors' model both of these features increase the probability of agrowth positions. It is therefore possible that the differences between researchers and the general public are also driven by demographic differences. It would be more interesting to compare researchers with more comparable demographics.

We appreciate the reviewer's comment and want to make three clarifications:

1. *On the issue of potential overfitting:*
    a. *While our models include 53 covariates (including country fixed effects), we have taken steps to ensure that overfitting does not bias the results. Specifically, already in the original submission we tested for multicollinearity using Variance Inflation Factors (VIF), and all values were well below the conservative threshold of 5, indicating that the predictors are not collinear (reported in the Methods section).*
    b. *To further probe the risk of overfitting, in the revised manuscript we tested the Least Absolute Shrinkage and Selection Operator (LASSO) to confirm that the key covariates*

highlighted in the discussion remain stable when penalizing less informative predictors. As we demonstrate in Table S10 in the Supplementary Information, the results obtained with Lasso confirm that our results are robust.

c. Importantly, our results are based on a very large sample (n = 16,781), which makes the model well-powered to handle a relatively large number of covariates without overfitting concerns.

2. On the difference between OLS and multinomial regression interpretation. We employ two complementary approaches:

The OLS model with the Growth Support index (GS) provides a continuous measure ranging from -1 (growth-sceptical) to +1 (pro-growth strong). This collapses the four opinion clusters into a single dimension and captures overall tendencies. The multinomial regression instead compares the probability of belonging to one of the three opinion clusters relative to the baseline category ("pro-growth moderate"). This allows us to identify how covariates shift respondents between specific positions but does not assume a simple linear continuum.

This explains why, for example, education shows up as positively associated with the GS index (implying a general tendency towards stronger growth support), while in the multinomial model, education mainly predicts a lower likelihood of being in the growth-sceptical group relative to pro-growth moderate, without affecting all contrasts equally. Similarly, gender and urban residence influence cluster membership in ways that do not translate into significant shifts in the GS index.

In short, the OLS provides a broad measure of directional support for growth, while the multinomial model highlights categorical distinctions between clusters. The fact that some variables are significant in one model but not the other is consistent with the fact that the two methods capture different aspects of the underlying distribution of opinions. We clarify this in the Methods section of the revised manuscript.

3. As for the sociodemographic features, we believe there is a potential confusion being made. All the analyses starting from Figure 1 (former Figure 2) till Figure 5 (former Figure 6) are done exclusively based on the general public sample across the 13 European countries. Survey data for researchers was only used in Figure 6 (former Figure 1) for comparison purposes. We have now added a clarification on this in the revised manuscript to avoid possible confusion.

Regarding the survey among researchers, we tend to agree that researchers are by default more educated and are more likely to live in urban areas. However, the purpose of Figure 6 is to compare a representative sample of society as a whole to a particular subset of society (i.e., climate policy researchers), which will naturally lean towards certain sociodemographics. We have added a caveat about this in the discussion section for readers to consider when interpreting the results.

4. Limited Causal Links and Theoretical Scope. In its current form, the analysis is very descriptive in nature. I am convinced the data at hand could be leveraged for clearer causal identification strategy that would help to form clearer policy recommendations

The nature of the data we use is non-experimental and cross-sectional, which means we can only derive associational or correlational results. We clarify this limitation now in the Methods section of the revised manuscript. We also make sure our results are interpreted by us only as correlations and not causal links.

Response to reviewer #2

I co-reviewed this manuscript with one of the reviewers who provided the listed reports. This is part of the Nature Communications initiative to facilitate training in peer review and to provide appropriate recognition for Early Career Researchers who co-review manuscripts.

Response to reviewer #3

(Our response is in Italics)

This paper offers a valuable empirical contribution by examining public attitudes toward economic growth and their alignment with climate policy preferences across 13 EU countries. Based on a large, representative survey and robust analytical techniques (notably Latent Class Analysis and regression modeling), it provides a nuanced typology of growth attitudes—distinguishing between strong/moderate pro-growth, agrowth, and degrowth orientations—and compares these with expert opinion. The data are rich, the methodology is transparent, and the results are potentially highly relevant to ongoing debates in sustainability and political economy.

Thank you for your positive evaluation and suggestions to improve the quality of our paper.

That said, several areas require further development to meet the standards of a journal such as NC. Most notably, the conceptual framing of "growth" remains under-theorized. The categories of green growth, agrowth, and degrowth are introduced descriptively, but the paper does not engage deeply with the normative, political, or epistemological tensions underlying these positions. This limits the interpretive depth of the findings, especially where the authors highlight surprising correlations—such as the positive association between pro-growth views and values like benevolence and equality—without probing whether respondents interpret "growth" in instrumental, moral, or nationalistic terms.

We appreciate this comment and agree that our initial discussion did not sufficiently address the broader normative, political, and epistemological tensions surrounding the academic debate on economic growth. In response, and in with feedback from another reviewer, we have revisited both the overall framing of the paper and the labels used for the clusters. We now frame the analysis more explicitly around how citizens view the importance of growth for achieving a stable and sustainable economy, which is a question that is more directly addressed by our four survey items. We have also changed the labelling of "agrowth" to "growth-indifference" and "degrowth" to "growth-scepticism". Within this revised framing, the positive correlations between pro-growth views and values like benevolence and equality can be more easily understood as citizens perceiving economic growth as a means to achieve inclusive societal wellbeing, which we now discuss in the revised paper. We also extend the discussion to reflect on how these public understandings may diverge from the more critical framings of economic growth that are common in academic debates.

In addition, the analysis at times blurs the line between correlation and causation. For example, the claim that political trust leads to stronger growth support is not supported by the cross-sectional design and should be more cautiously stated. The role of gender, climate concern, and lifestyle proxies (e.g., flight frequency) in shaping growth attitudes is reported but insufficiently theorized. These findings could benefit from engagement with existing literature on gendered environmental attitudes or psychological mechanisms like motivated reasoning.

We have carefully double-checked the manuscript to ensure that we interpret our results as strictly correlational, and not causal. Regarding gender, climate concern, and lifestyle proxies: in the OLS regression with the Growth Support Index, none of these variables showed significant relationships. Statistically significant results only appeared in the multinomial regression of

opinion clusters, but these patterns are more nuanced and difficult to theorise. For example, growth-sceptical respondents were more likely to be male than the reference pro-growth moderate cluster, while growth-indifferent respondents were more likely female than the reference cluster; climate concern was higher among both growth sceptical and pro-growth strong respondents than among the reference cluster. These results present nuanced pictures of differences between these clusters of growth opinions, which we are cautious to conjecture about. Given the number of factors included in the overall analysis, we also do not want to overinterpret results for effects that are not intuitive and small.

As our dependent variable is growth support and not environmental concern, these patterns cannot be straightforwardly linked to the literature on gendered environmental attitudes. It appears that men are more likely to be drawn to a more radical form of post-growth (i.e. growth sceptical) than women (more likely to be growth-indifferent). However, we cannot infer that the motivation for the more radical views among men is related to environmental attitudes (and we are indeed controlling for climate concern). In the revised manuscript we therefore present these findings as raising novel questions for future research into the sociopsychological foundations of growth attitudes rather than as evidence for existing theories.

Finally, while the comparison to expert surveys is a useful framing device, the implications of the public–academic gap are underdeveloped. What does this misalignment suggest about the prospects for democratic legitimacy or effective climate governance? The discussion gestures at policy implications but remains general.

One key implication of our results is that preferences for growth may mean different things for experts and the general public. Based on experts' views about growth, preferences for growth are easily interpreted as prioritising the economy and self-interest over sustainability. However, this interpretation should not be applied to the general public, as many see economic growth as essential to achieving diverse sustainability goals. In fact, our results suggest that a strong preference for economic growth among the general public – for instance, reflected in voting behaviours and policy support – may originate from a strong concern about sustainability, rather than not caring about this, despite the latter often being suggested.

There are different views to interpret the public–academic gap. One view is that it underscores the importance of improving the translation of scientific expertise into public discourse to ensure voter preferences are better informed about the relationship between climate policy and growth objectives. Another view is that such convergence may be difficult to achieve in practice, given that these issues have already been extensively debated in the public sphere and remain contested. From this perspective, the gap raises questions about the democratic legitimacy of post-growth ideas and their political feasibility. We agree these considerations were lacking in the previous version of the manuscript, so we have added text to the discussion to reflect both interpretations.

In sum, the paper is empirically strong and timely, but would benefit from tighter conceptual framing, more careful interpretation of associations, and deeper engagement with the theoretical stakes of its findings. A revised version with these improvements would be a strong candidate for publication.

Thank you for this positive assessment.

Reviewer #3 (Remarks on code availability):

The link led to a GitHub site "404 not available"

Sorry for the inconvenience, the repository on GitHub was set as private but has been made accessible now. https://github.com/IvanVSavin/Growth-optimism-in-the-EU

Response to reviewer #4

(Our response is in Italics)

The paper explores public attitudes towards (green) growth and post-growth in light of climate change. This is certainly an important topic to study and such the paper could make an interesting contribution to the literature. The main value-added / novelty is the rich 19,000 responses-strong survey in 13 EU countries. The statistical manipulations to analyze those data are relatively straightforward.

Thank you for your positive evaluation and suggestions to improve the quality of our paper.

I have the following observation to strengthen the manuscript before it is ready for publication.

1. Definitions of growth paradigms. Unlike other papers, this survey did not ask people directly about their views on different notions of growth. Instead, attitudes are derived from the answers to four proxy questions. I'm not sure the proxy answers map neatly to the usual growth paradigms found in the literature. For example, disagreeing with the statement "economic growth is necessary to finance environmental protection" is not the same as agreeing with the statement "economic growth is incompatible with environmental protection" and only the latter is an unequivocal degrowth position. Perhaps I disagree with the statement because I think we can already afford environmental protection, without having a view on (de)growth. Similarly, the statements about public services and stability are statements about conventional economic growth, rather than green growth (and indeed the green growth label is not consistently used). Or, as a final example, respondents who are neutral to the four statements may register a "don't know" answer or subscribe to notions of weak green growth ("we can decouple economic activity from environmental damage"; Jacobs 2013) rather than being growth-agnostic. The authors should review the labels they assign to the various positions.

Our approach of inferring growth positions from attitudinal rather than direct statements follows an established line of work in the literature. Drews & van den Bergh (2016) combined asking respondents direct questions about their growth-vs-environment preferences and conducted a factor analysis of 22 additional statements to examine the structure of attitudes towards economic growth. Building on this, Drews et a. (2019) used the items that identified green growth, agrowth, and degrowth clusters, which strongly correlated with the direct responses. Savin et al. (2021) then demonstrated that a reduced set of four statements was sufficient to approximate these clusters with high accuracy, making the instrument suitable for larger surveys where space is limited. This even distinguished between specific question sets for citizens and researchers. While this approach naturally has its limitations, asking respondents to choose one of four distinct positions also risks oversimplifying complex attitudes and is highly sensitive to framing effects, which are issues we discussed when explaining our choice of survey instrument in the manuscript.

Nonetheless, we generally agree with your broader point that, when taken in isolation, the survey instrument does not directly map to the growth-vs-environment paradigms discussed in the literature. We have therefore revisited the framing of the paper and the labels we assign to the clusters. Instead, we frame the paper around citizens' attitudes towards economic growth to achieve a stable and sustainable economy.

While we had already shifted our labelling of the clusters away from "green growth" to "pro-growth" in the original manuscript, some references (most noticeably in Figure 2) still used the earlier framing. We have now carefully revised the manuscript to ensure that the "pro-growth" (and not "green growth") framing is applied consistently.

The cluster previously called "agrowth" has been relabelled to "growth-indifferent" and the "degrowth" cluster to "growth-sceptical". As an additional point on the growth-indifferent cluster, we clarify that respondents were given a "don't know" option for each of the four statements. Any respondent who answered "don't know" to at least one of them was removed from the sample. While this was already noted in the Methods, we have now also highlighted this in the main text to aid interpretation.

We have also reordered the paper so that the results for citizens are reported first and then later compared to the results for climate policy researchers, where we discuss how the findings for citizens correspond to the academic debate while acknowledging the strong correlation between the survey instrument we used with the three main positions distinguished in the literature.

2. Statistical model. I am not a statistician, but was wondering about the inclusion of of the policy instruments variables in the main regressions. These strike me as endogenous / correlated with other covariates. For example, if I had to explain ETS support, I would include variables like climate concern, trust and the socio-demographic variables. This might then suggest eg a two-stage least square approach. A corollary of this technical point is that the correlations shown in Figure 6 may simply reflect the fact that both growth attitudes and policy preferences are shaped by the same socio-economic factors.

We appreciate the reviewer's comment on the inclusion of policy instrument variables. Our intention in incorporating these variables was not to estimate causal effects, but rather to examine whether support for specific climate policies correlates with citizens' positions on growth. In the revised manuscript, we clarify that our analysis should be interpreted as correlational but not causal.

We agree that policy support is itself shaped by socio-demographics, trust, and climate concern. In fact, when these variables are omitted, some policy measures (e.g. ETS for transport and heating) appear significant in simple regressions (see Tables S11–S13 in the Supplementary Information). This highlights that variation in policy preferences is partly explained by these underlying characteristics. By pooling socio-demographics, political views, values, and policy support in the same model, we are able to capture conditional correlations: that is, whether preferences for particular policy instruments remain systematically associated with growth attitudes once these other observable factors are accounted for.

3. Researcher vs public attitudes. The comparison of public attitudes to those of researchers is interesting, but a few caveats may be required. Even if the questions were exactly the same (which I assume to be the case), the authors are comparing a 2021 researcher survey with a 2024 public survey. Difference may be due either to the different respondents or the different timeframe. Most notably, the Ukraine war from 2022 onward and associated energy price hikes / economic fragility may well have affected growth attitudes.

We agree that there are some considerations that should be taken into account when comparing the results between citizens and researchers, particularly as a result of the events you mention occurring between the two surveys. These considerations have now been added to the manuscript as a caveat for comparing the findings between the two surveys.

4. A couple of smaller points

- p.10: why is the lack of correlation with climate concerns unexpected? It is perfectly consistent with green growth views (even if these are not captured very well by the four proxy questions see above).

We agree "unexpected" was not the best choice of words here. While it may suggest that those with "growth-at-all costs" opinions form a minor proportion of those with pro-growth views, this is consistent with previous citizen surveys, so it is not really unexpected. This point has been clarified in the text, and the reference to it being "unexpected" has been removed.

p.15: why is urban residence a proxy for a high-carbon lifestyle? City dwellers are more likely to use public transport for example. They may (or may not) be better off, but you control for income.

We agree that the relationship between urban residence and carbon intensity is complex, particularly when controlling for income. We have therefore removed the reference to urban residence as being a proxy for high-carbon lifestyles.

- p.18: it is obviously fine to rescale the ordinal responses from the original Likert scale. By the same token, it is unclear what is being gained from it. A 1-7 scale is as intuitive as a -1 to 1 one.

While we agree that rescaling from 1 to 7 to -1 to 1 is not strictly necessary, we find that it aids interpretation. The -1 to 1 scale allows us to frame results more intuitively, with -1 corresponding to a growth-sceptical position, +1 to a strong pro-growth position, and 0 indicating neutrality. This framing is especially helpful in Figure 2, where the rescaled values clearly convey that country averages reflect moderately pro-growth attitudes.

- Chart S1 in the Supplementary Material should be made self-explanatory. What has been analysed here (LCA presumably), what is the scale of the y axis, what do the various acronyms (cAIC etc) stand for.

The figure in question indeed reports the model fit criteria from our Latent Class Analysis (LCA), which we used to determine the optimal number of clusters. The y-axis shows the values of different information criteria, and the acronyms correspond to standard statistical measures of model fit: BIC (Bayesian Information Criterion), cAIC (consistent Akaike Information Criterion), and aBIC (sample-size adjusted BIC). In line with best practice, we selected the four-cluster solution because it minimized the above-mentioned criteria.

This information has now been added to the caption of the Figure S1 in the Supplementary Materials to make it self-explanatory.

Response to reviewers

Manuscript Number: NCOMMS-25-33850A

Views of EU citizens on economic growth and implications for climate policy

Response to reviewer #4

(Our response is in Italics)

I continue to believe that this is an interesting empirical paper that sheds important light on public attitudes about growth in Europe.

The authors have responded thoughtfully and comprehensively to reviewer comments. Mostly this was achieved by changes to interpretations (e.g. emphasis on correlation vs causality) and framing (e.g. the growth labels) and by adding further explanations (e.g. on sampling strategy), rather than undertaking different analysis, although there are added statistical tests (e.g. LASSO).

I still believe the paper would be stronger without the section that compares the public survey with the researcher survey results. This comparison is dubious for the reasons set out by this and other reviewers before. These are now set out, but it begs the question why the paper contains a comparison that is explicitly acknowledged to be problematic.

We appreciate the reviewer's concern and agree that comparisons between citizens and researchers require careful interpretation. For this reason, the comparison in our manuscript was framed as illustrative. It is used to situate citizens' growth views relative to the expert discourse that motivates much of the post-growth debate in climate policy. This approach is not uncommon in the literature (e.g., Drews et al., 2019; Pearson and Wagner, 2025; Luis et al., 2020; Bebe et al., 2019; Sapienza and Zingales, 2013).

However, in response to the reviewer's concern, we have now moved this comparison from the main text to the Supplementary Information and briefly refer to it in the Discussion section. This ensures that the manuscript is focused on the citizen survey results while still allowing interested readers to see the comparison.

Beebe, J. R., Baghramian, M., Drury, L., & Dellsén, F. (2019). Divergent perspectives on expert disagreement: Preliminary evidence from climate science, climate policy, astrophysics, and public opinion. Environmental Communication, 13(1), 35-50.

Luís, S., Lima, M. L., Poggio, L., Aragonés, J. I., Courtier, A., Roig, B., & Blanchard, C. (2020). Lay people and experts' risk perception of pharmaceuticals in the environment in Southwestern Europe. Regulatory Toxicology and Pharmacology, 117, Article 104783. https://doi.org/10.1016/j.yrtph.2020.10478

Pearson, M., & Wager, A. (2025). Not so different: Comparing British MPs' and voters' attitudes to climate change. Parliamentary Affairs, 78(1), 53-76.

Sapienza, P., & Zingales, L. (2013). Economic experts versus average Americans. American Economic Review, 103(3), 636-642.

Response to reviewer #5

(Our response is in Italics)

This manuscript analyzes public opinion about economic growth using original data collected in 13 European countries, complemented with a survey of experts in multiple European countries. I agree with the authors' argument that public (and experts') opinions about the relation between environmental protection and economic growth is often more complex than a simple trade-off between the two and I also appreciate that they have invested a lot of efforts in terms of data collection and analysis.

Thank you for this positive evaluation.

However, even after the revision the paper continues to have several major weaknesses due to which I am unable to recommend it for publication.

First, the paper still lacks strong theoretical framework. The post-growth paradigm is discussed rather superficially. There is also no discussion of how socio-demographics, human values, trust, political ideology, specific policy preferences, etc. are expected to correlate with their dependent variable. While I acknowledge that the paper is exploratory and thus does not necessarily need to have testable hypotheses, there needs to be a justification why those independent variables might matter; otherwise, the reader gets an impression that the authors simply used any variable they had at hand. This also makes it difficult to interpret the results, many of which are quite surprising – e.g. more educated and universalist people being more likely to support growth, weak correlation between growth skepticism and environmental concern or environmental policy support, etc. – and post-hoc explanations/speculations are not particularly convincing.

Thank you for raising these points. We have taken them on board and believe this has resulted in a more focussed and coherent paper. In response to your feedback, we have substantially revised the introduction and discussion sections to provide a stronger theoretical framework and clearer framing of our study. In particular, we now clarify what sets our approach apart from previous studies: rather than implicitly framing a trade-off, we examine whether citizens perceive growth as an instrumental tool to achieve environmental and social goals. This framing helps contextualise some of our more surprising results, such as why individuals with universalist values might still support economic growth (e.g., because they view it as a necessary for financing sustainability). Additionally, we have strengthened the motivation for our independent variables at the beginning of each sub-section in the results section to ensure the rationale for their inclusion is clear and theoretically grounded.

Second, the authors cannot seem to make up their mind whether their dependent variable is continuous or nominal. Based on their argument about the complexity of opinions about growth vs. environment, and also the literature they cite (e.g. several papers by Van Den Bergh, Savin and Drews), segmentation approach seems more appropriate, so personally I would opt for clustering, but if they prefer a continuum, they should justify why. In any case, the two approaches are incompatible and cannot be used simultaneously, even for robustness test.

We appreciate the reviewer's concern regarding the simultaneous use of a continuous measure and discrete opinion clusters, and we agree that the choice of representation requires clear justification. However, we do not consider these two approaches to be conceptually or

empirically incompatible. Rather, they capture different but complementary aspects of the same underlying attitude structure. A continuous measure captures the intensity of support or opposition along a single dimension, while clustering or segmentation identifies qualitatively distinct response profiles and potential non-linearities that are not well represented by a single scale.

This practice is particularly well established in environmental attitude research. A prominent example is the "Six Americas" literature on climate change opinions. In this work, respondents are segmented into discrete opinion groups using clustering or latent class approaches (e.g. Alarmed, Concerned, Cautious, etc.), analysed using multinomial logit models to assess socio-demographic and attitudinal correlates. At the same time, these studies employ continuous indices of climate concern, risk perception, or policy support, which are analysed using OLS or related regression techniques. Importantly, the segments are explicitly interpreted as based upon an underlying continuum of concern, and the two representations are treated as complementary rather than competing (Chryst et al., 2018). Similar combined use of categorical and continuous representations can be found more broadly in the study of environmental concern and sustainability attitudes. For example, Poortinga et al. (2004) analyse environmental concern using continuous scales while also distinguishing between different attitudinal profiles, showing that the two approaches highlight different but consistent aspects of the same underlying belief structure.

Within the growth-versus-environment literature specifically, Drews et al. (2019) identify discrete opinion clusters among both citizens and researchers but explicitly interpret these clusters as ordered along a spectrum from pro-growth to growth-sceptical views. Their analysis demonstrates that segmentation does not preclude an underlying continuum; rather, the two perspectives are analytically complementary. Our approach follows this tradition. The Growth Support Index provides a parsimonious summary of overall support intensity based on the same four survey items, while the multinomial analysis of clusters highlights differences between opinion profiles.

We have therefore retained both approaches in the revised manuscript, but now make clear at the beginning of the Results section the justification for doing so and that they are used for descriptive completeness rather than as alternative or competing approaches.

Chryst, B., Marlon, J., van der Linden, S., Leiserowitz, A., Maibach, E., & Roser-Renouf, C. (2018). Global Warming's "Six Americas Short Survey": Audience segmentation of climate change views using a four-question instrument. Environmental Communication, 12(8), 1109–1122. https://doi.org/10.1080/17524032.2018.1508047

Drews, S., Savin, I., & van den Bergh, J. C. J. M. (2019). Opinion clusters in academic and public debates on growth versus environment. Ecological Economics, 157, 141–155. https://doi.org/10.1016/j.ecolecon.2018.11.012

Poortinga, W., Steg, L., & Vlek, C. (2004). Values, environmental concern, and environmental behavior. Environment and Behavior, 36(1), 70–93. https://doi.org/10.1177/0013916503251466

Third, related to the previous point, the fact that the authors identify four clusters (as opposed to three clusters identified by Van Den Bergh and Drews) is problematic as 'pro-growth strong'

and 'pro-growth moderate' clusters both correspond to a single 'green growth' position. I acknowledge that the choice of four clusters is statistically correct (judged by lowest cAIC and BIC), but substantively it makes more sense to treat them as a single group as both support growth, even if with different intensity.

First, we would like to clarify that prior studies by Drews and van den Bergh never found only three clusters in the growth-vs-environment debate. Drews and van den Bergh (2016), using a sample of the general public in Spain, found four clusters (the fourth one was "growth at all costs"). The same result, though with different numerical shares, was obtained by Bell et al. (2025). Another study by Drews and van den Bergh (2017), undertaking an international survey of scientists, found five clusters. Drews et al. (2019) using Latent Class Analysis found four clusters among general public in Spain. A study by McNeil and Barnes (2025) also employing Latent Class Analysis to elicit views on growth-vs.-environment trade-off reports six different clusters. This demonstrates that the growth-vs-environment debate is more nuanced and cannot be always captured in three opinions.

We agree that both the "pro-growth strong" and "pro-growth moderate" clusters fall on the pro-growth side of the spectrum. However, the purpose of identifying four clusters is not to introduce additional conceptual paradigms beyond those discussed in the literature, but to capture heterogeneity in the intensity and structure of pro-growth beliefs within the general public. Treating all pro-growth respondents as a single group would obscure substantively meaningful differences that are relevant for understanding policy preferences and value associations. Note that the two clusters combined account for almost 60% of the population, and instead of forcing them into one cluster, it is worthwhile to see the differences among them.

Empirically, the four-cluster solution is supported by standard information criteria, but more importantly, the two pro-growth clusters differ systematically in their responses across all four growth statements as well as in their associations with socio-demographics, values, and climate policy preferences. This pattern indicates that the distinction is not merely one of arbitrary scale cut-offs but reflects qualitatively different response profiles. Similar distinctions between strong and moderate supporters within a broadly shared orientation are common in public opinion research and are often crucial for explaining differences in policy support and political behaviour. This is now better explained in the paper.

Substantively, we interpret the two pro-growth clusters as sub-types within a single pro-growth orientation, rather than as distinct paradigms comparable to agrowth or degrowth. This is why we label them "strong" and "moderate" pro-growth and avoid equating them with separate growth paradigms. This approach is consistent with segmentation practices in environmental attitude research, where groups are frequently distinguished by degree of support or engagement even when they share the same directional position (e.g. Leiserowitz et al., 2010; Chryst et al., 2018).

To avoid any potential misunderstanding, we have clarified in the revised manuscript that the key conceptual contrast remains between pro-growth, growth-indifferent, and growth-sceptical views, and that the split between strong and moderate pro-growth captures within-group heterogeneity rather than additional paradigmatic positions.

Chryst, B., Marlon, J., van der Linden, S., Leiserowitz, A., Maibach, E., & Roser-Renouf, C. (2018). Global Warming's "Six Americas Short Survey": Audience segmentation of climate change views using a four-question instrument. Environmental Communication, 12(8), 1109–1122. https://doi.org/10.1080/17524032.2018.1508047

Leiserowitz, A., Maibach, E., Roser-Renouf, C., & Smith, N. (2010). Climate change in the American mind: Americans' global warming beliefs and attitudes in January 2010. Yale and George Mason University. Yale Project on Climate Change.

McNeil, A., & Barnes, L. (2025). The environment–economic growth trade-off: does support for environmental protection depend on its economic consequences? Ecological Economics, 230, 108522.

Fourth, given that the data is available for 13 countries only, which is too few for multi-level modelling, I do not think that the substantive claims can be made about the effects of contextual variables, such as affluence and inequality. Reporting correlations with 13 cases should definitely be avoided. Instead, I would suggest using country fixed-effects models and then also replicating pooled data results at country level for a robustness test to make at least some use of cross-national nature of the data (in addition to reporting country-level distribution of clusters in Figure 1 which is completely blurry in the pdf).

We agree that, considering the number of observations (countries), we should refrain from statistical inference at the country level and present these associations as contextual patterns that help situate the individual-level results, not as standalone evidence. We would like to mention, however, that reporting descriptive country-level correlations with a small number of country cases (e.g., ~10–30) is common when the goal is to illustrate macro-level patterns (and their plausible alignment with micro-level results) rather than to conduct country-level hypothesis tests, provided that the manuscript avoids over-interpreting these. For instance, White and Muthukrishna (2024) analyze value divergence across World Values Survey waves and report country-level associations/regression coefficients relating "value distance/similarity" to geopolitical covariates. They note that some wave-specific analyses involve small country Ns (e.g., n = 11 at one early time point and n = 20 at another), and they treat these relationships as contextual patterns rather than definitive causal estimates.

Likewise, Luo et al. (2025) explicitly relate cross-national effect patterns to country-level moderators using a multi-country dataset (23 countries) and report that the association of Internet use with mental-health outcomes is correlated with country-level factors (e.g., inequality/digital-skills indicators) in supplementary analyses. This again illustrates a standard "macro-context" step that complements, rather than substitutes for, individual-level modelling. Finally, Seijmonsbergen-Schermers et al. (2020) compare intervention rates across 13 countries and directly examine country-level correlations between intervention rates (and between interventions and outcomes), highlighting cross-country comparability limits and using the correlations to describe system-level patterns rather than to claim country-level causal effects. To address this concern, we have now clarified in the manuscript that the country-level analysis is descriptive and illustrative.

At the individual level, all regression analyses already include country fixed effects (see Supplementary Information), ensuring that the reported associations between growth views and individual characteristics are identified from within-country variation only. We have clarified it now also in the revised version of the manuscript. Thus, none of our main regression results relies on cross-national variation or contextual variables. We now clarify this in the revised manuscript. In the Supplementary Information in Table S14 we now report results obtained separately for Denmark, Germany, and Spain using least squares model and growth support index. As one can

see while there are some moderate differences across countries, most of our results emphasized in the main text are robust.

Finally, we agree that clearer visual presentation is important. We have therefore improved the readability of Figure 1 in the revised manuscript to ensure that cross-country differences in cluster distributions are clearly visible.

Luo, Y., Yip, P. S. F., & Zhang, Q. (2025). Positive association between Internet use and mental health among adults aged ≥50 years in 23 countries. Nature Human Behaviour, 9(1), 90–100. https://doi.org/10.1038/s41562-024-02048-7

Seijmonsbergen-Schermers, A. E., van den Akker, T., Rydahl, E., Beeckman, K., Bogaerts, A., Binfa, L., et al. (2020). Variations in use of childbirth interventions in 13 high-income countries: A multinational cross-sectional study. PLOS Medicine, 17(5), e1003103. https://doi.org/10.1371/journal.pmed.1003103

White, C., & Muthukrishna, M. (2024). Worldwide divergence of values. Nature Communications, 15, 2650. https://doi.org/10.1038/s41467-024-46581-5
